# Combination of angiotensin-(1–7) with perindopril is better than single therapy in ameliorating diabetic cardiomyopathy

**DOI:** 10.1038/srep08794

**Published:** 2015-03-05

**Authors:** Panpan Hao, Jianmin Yang, Yanping Liu, Mingxiang Zhang, Kai Zhang, Fei Gao, Yuguo Chen, Cheng Zhang, Yun Zhang

**Affiliations:** 1 The Key Laboratory of Cardiovascular Remodeling and Function Research, Chinese Ministry of Education and Chinese Ministry of Public Health, Qilu Hospital, Shandong University, Jinan 250012, Shandong, China; 2 Shandong Provincial Key Laboratory of Diagnosis and Treatment of Cardio-cerebral Vascular Diseases, Shandong Medical Imaging Research Institute, Shandong University, Jinan 250021, Shandong, China

## Abstract

We recently found that overexpression of angiotensin (Ang)-converting enzyme 2, which metabolizes Ang-II to Ang-(1–7) and Ang-I to Ang-(1–9), may improve left ventricular remodeling in diabetic cardiomyopathy. Here we aimed to test whether chronic infusion of Ang-(1–7) can dose-dependently ameliorate left ventricular remodeling and function in a rat model of diabetic cardiomyopathy and whether the combination of Ang-(1–7) and Ang-converting enzyme inhibition may be superior to single therapy. Our results showed that Ang-(1–7) treatment dose-dependently ameliorated left ventricular remodeling and dysfunction in diabetic rats by attenuating myocardial fibrosis, myocardial hypertrophy and myocyte apoptosis via both the Mas receptor and angiotensin II type 2 receptor. Furthermore, combining Ang-(1–7) with perindopril provided additional cardioprotection relative to single therapy. Ang-(1–7) administration provides a novel and promising approach for treatment of diabetic cardiomyopathy.

Diabetic cardiomyopathy (DCM), characterized by left ventricular (LV) remodeling and dysfunction, is associated with substantial risk of heart failure and increased mortality^1^. Cardiac fibrosis, myocardial hypertrophy and myocyte apoptosis, are considered the three major pathological features of the LV remodeling in DCM^2^. Of particular importance is the extensive accumulation of interstitial collagen, a hallmark of LV remodeling in DCM with reduced myocardial contractility and increased LV stiffness.

A wealth of evidence indicates that the renin-angiotensin system (RAS) plays an important role in the pathogenesis of DCM^3^, and angiotensin-converting enzyme (ACE), angiotensin II (Ang-II), angiotensin-converting enzyme 2 (ACE2) and angiotensin-(1–7) [Ang-(1–7)] are considered important components of RAS. Previous studies have demonstrated that ACE inhibitors and Ang-II type 1 receptor (AT_1_R) antagonists are efficacious in improving LV remodeling and function in DCM. Recently, we and others found that ACE2 exerted cardioprotection in diabetic and other models via downregulating Ang-II and upregulating Ang-(1–7) levels^4^,^5^,^6^,^7^,^8^. Similarly, Ang-(1–7) attenuated LV remodeling and dysfunction induced by myocardial infarction or hypertension^9^,^10^. A recent study reported that Ang-(1–7) completely rescued the diastolic dysfunction in db/db diabetic mice, but the effect on LV remodeling and systolic dysfunction was unclear because db/db hearts showed preserved LV volume and systolic function^11^. Moreover, in another study, Ang-(1–7) infusion did not reverse the systolic dysfunction in ACE2-deficient diabetic Akita mice^4^.

Ang-(1–7), a heptapeptide converted from Ang-II by ACE2, binds to a distinct plasma membrane G protein-coupled receptor, the Mas receptor (MasR), and exerts vasodilative, anti-proliferative and anti-inflammatory effects. Our recent study found that ACE2 overexpression downregulated AT_1_R protein expression *in vivo* and *in vitro*, which suggests that the effects of Ang-(1–7) may involve receptors other than MasR^12^. Moreover, as ACE inhibition may reduce Ang-II generation and inhibit conversion of Ang-(1–7) into inactive Ang-(1–5)^13^, combining exogenous Ang-(1–7) with ACE inhibitors might provide more cardioprotection than single therapy. Thus, several important issues raised by recent studies need to be clarified. The first issue is whether there is a dose-effect relationship between Ang-(1–7) and DCM, whether Ang-(1–7) is superior to ACE inhibition in the treatment of DCM and whether the combination of Ang-(1–7) and ACE inhibition is better than Ang-(1–7) alone in alleviating DCM. In addition, we wondered about the roles of AT_1_R, Ang-II type 2 receptor (AT_2_R) and MasR in mediating the therapeutic effects of Ang-(1–7) in DCM. To address these issues, we examined *in vivo* and *in vitro* whether chronic infusion of Ang-(1–7) may dose-dependently ameliorate LV remodeling and function in a rat model of DCM, and whether Ang-(1–7) and ACE inhibition combined may be superior to single therapy.

## Methods

Please see the Online Appendix for details.

### Ethics statement

All experiments were performed in accordance with the Guide for the Care and Use of Laboratory Animals published by the US National Institutes of Health (NIH Publication, 8th Edition, 2011). The Institutional Animal Care and Use Committee at Qilu Hospital, Shandong University approved the experiments.

### Animal model

We divided 126 male Wistar rats into 2 groups: DCM model (n = 112) and control (n = 14). Diabetes was induced by a single intraperitoneal injection of streptozotocin. At the end of week 12 after injection, all rats in the DCM model group were again divided into 8 groups for treatment: mock, perindopril, low-dose Ang-(1–7), moderate-dose Ang-(1–7), high-dose Ang-(1–7), high-dose Ang-(1–7) + perindopril, high-dose Ang-(1–7) + A779 (a MasR antagonist) and high-dose Ang-(1–7) + PD123319 (an AT_2_R antagonist).

### Blood pressure and blood glucose measurement

Heart rate, systolic blood pressure, diastolic blood pressure and mean arterial pressure were measured before and after treatment by use of a noninvasive tail-cuff device (Softron BP-98A; Softron, Tokyo) as described previously^2^. Fasting blood glucose level was analyzed by use of the Bayer 1650 blood chemistry analyzer (Bayer, Tarrytown, NY).

### Echocardiographic and hemodynamic measurement

Echocardiographic and hemodynamic measurement was performed before and after treatment as described^2^,^5^ with modifications.

### Histology

We used 4-μm paraffin-embedded tissue sections for hematoxylin and eosin and Masson trichrome staining to assess tissue architecture and interstitial and perivascular fibrosis.

### Transmission electron microscopy (TEM)

After hearts were excised, fresh LV tissue was quickly cut into 1-mm cubes and underwent standard block preparation for TEM.

### Real-time RT-PCR

The mRNA levels of genes were determined as described^2^ and their relative levels were quantified by the 2^−ΔΔCT^ method, with β-actin as the endogenous reference gene. Primer sequences are listed in Supplementary Table 1.

### Ang-(1–7), angiotensin-(1–9) [Ang-(1–9)] and Ang-II levels

Ang-(1–7), Ang-(1–9) and Ang-II levels were determined by use of HPLC-based radioimmunoassay as described^14^.

### ACE and ACE2 activities

ACE and ACE2 activities were determined with assays based on internally quenched fluorescent substrates.

### Activity of a disintegrin and metalloproteinase 17 (ADAM17)

Activity of ADAM17 (also called tumor necrosis factor-α–converting enzyme), was determined by use of the SensoLyte 520 ADAM17 Activity Assay Kit Fluorimetric (AnaSpec, San Jose, CA).

### Isolation and culture of neonatal rat cardiac fibroblasts and myocytes

Neonatal rat cardiac fibroblasts and myocytes were isolated and cultured as described^15^ with modification.

### ^3^H-proline incorporation assay

Collagen synthesis of cultured cardiac fibroblasts was measured by ^3^H-proline incorporation as described^5^ with modifications.

### ELISA

ELISA was used to measure protein levels of soluble collagen I and III and transforming growth factor (TGF)-β1 in the medium of cardiac fibroblasts.

### Assessment of cardiomyocyte hypertrophy

The cross-sectional area of myocytes in cardiac sections was measured by staining with Alexa Fluor 488-conjugated wheat germ agglutinin (Invitrogen, Carlsbad, CA) and the surface area of cultured cardiomyocytes was determined by immunostaining with a rabbit polyclonal antibody against myosin heavy chain (Santa Cruz Biotechnology, Santa Cruz, CA).

### Detection and quantitation of apoptosis

Apoptotic cells in tissue sections were quantified by terminal deoxynucleotidyltransferase–mediated dUTP nick-end labeling (TUNEL). Apoptosis of cultured cardiomyocytes was evaluated by double immunofluorescence for myosin heavy chain and TUNEL.

### Dihydroethidium fluorescence and lucigenin-enhanced chemiluminescence

The oxidative fluorescent dye dihydroethidium was used to measure superoxide (O_2_^−^) levels in myocardial frozen sections and cultured cardiac fibroblasts and myocytes^8^. NADPH oxidase activity in myocardial tissues and cardiac fibroblasts and myocytes was quantified by lucigenin-enhanced chemiluminescence as describe8.

### Western blot, immunohistochemistry, and immunocytochemistry

Western blot, immunohistochemistry, and immunocytochemistry were performed with standard methods.

### Statistical analysis

SPSS v11.5 (SPSS Inc., Chicago, IL) was used for statistical analysis. Continuous data are expressed as mean ± SEM and compared by one-way ANOVA, followed by Tukey–Kramer post-hoc test and independent samples *t* test. *P* < 0.05 was considered statistically significant.

## Results

### Ang-(1–7) has no effect on blood glucose level

One week after streptozotocin injection, fasting blood glucose level was markedly elevated in the model group and remained high until the end of the experiment, and its level did not differ between the mock group and the 7 treatment groups at the end of weeks 12 (Supplementary Table 2) and 16 (Supplementary Table 3). No apparent side effects were observed in any treatment group.

### Ang-(1–7) prevents LV dysfunction

At 12 weeks after streptozotocin injection, rats in the mock group showed decreased LV ejection fraction, fractional shortening, E/A, E'/A', maximal LV systolic pressure and ±dp/dt and increased LV end-systolic diameter, LV end-diastolic diameter and LV end-diastolic pressure as compared with the control group (Supplementary Tables 4 and 5). These measurements were dose-dependently improved by 4-week treatment with Ang-(1–7), and the salutary effects were enhanced by co-administration of perindopril and largely offset by A779 or PD123319 (Supplementary Fig. 1 and Tables 1 and 2). However, Ang-(1–7) had no significant effect on blood pressure and heart rate in DCM rats (Supplementary Table 3).

### Ang-(1–7) improves LV ultrastructural abnormalities

**Figure 1:**
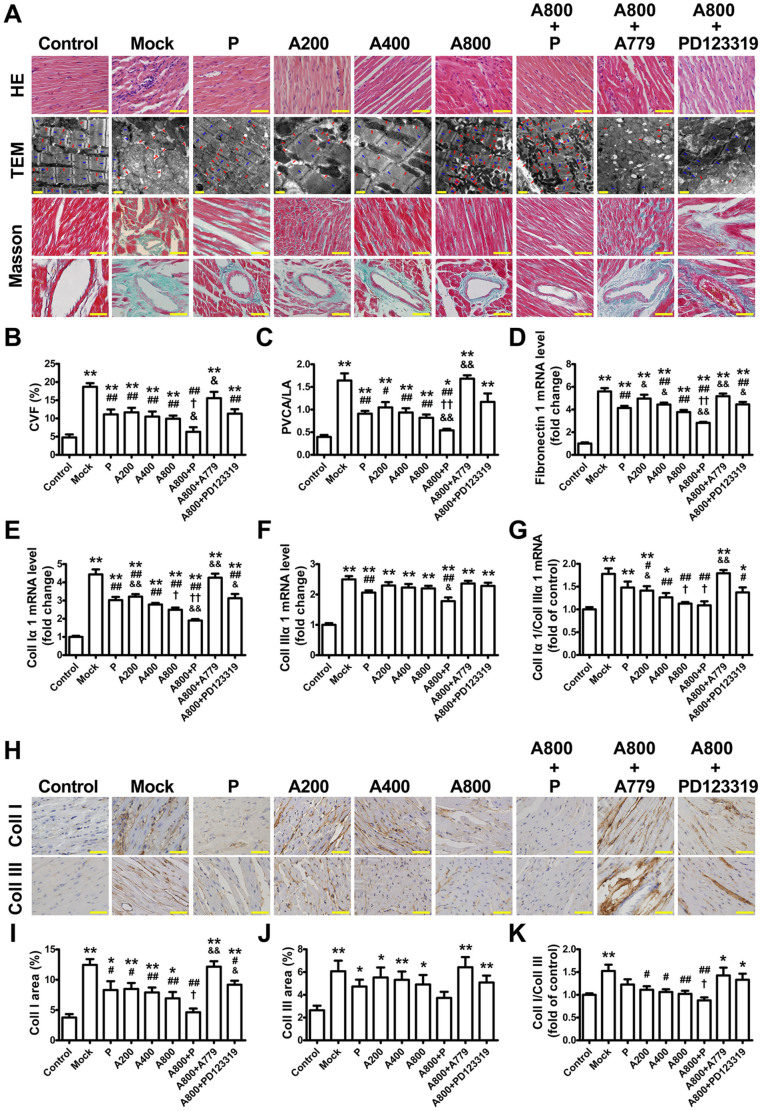
Myocardial fibrosis in 9 groups of rats. (A) Representative hematoxylin and eosin staining (top panel, H&E) (scale bar: 50 μm), representative transmission electron micrographic images (mid panel, TEM) showing regular striations of myofibrils with clear Z lines and more dense and less swollen mitochondria with Ang-(1–7) than mock treatment (scale bar: 0.5 μm; red arrows: mitochondria; blue arrows: Z lines), and representative Masson trichrome staining (collagen stained as green and myocardium as red; bottom panels, scale bar: 50 μm) showing interstitial and perivascular fibrosis. Quantitative analysis of collagen volume fraction (CVF) (B) and perivascular collagen area to luminal area (PVCA/LA) (C). Quantitative analysis of mRNA expression of fibronectin 1 (D), collagen type I-α1 (Coll Iα1) (E), collagen type III-α1 (Coll IIIα1) (F) and ratio of Coll Iα1 to IIIα1 (G). (H) Representative immunohistochemical staining for collagen I (Coll I) and III (Coll III) (scale bar: 50 μm). Quantitative analysis of Coll I (I) and Coll III (J) staining and ratio of Coll I to III (K). *P < 0.05 and **P < 0.01 vs. control; ^#^*P* < 0.05 and ^##^*P* < 0.01 vs. mock; ^†^*P* < 0.05 and ^††^*P* < 0.01 vs. perindopril (P); ^&^*P* < 0.05 and ^&&^*P* < 0.01 vs. high-dose Ang-(1–7) (800 ng·kg^−1^·min^−1^) (A800). A200 = low-dose Ang-(1–7) (200 ng·kg^−1^·min^−1^); A400 = moderate-dose Ang-(1–7) (400 ng·kg^−1^·min^−1^).

TEM revealed clear sarcomeres and Z lines and apparently normal-sized mitochondria, with normal numbers, in the LV myocytes of the control group but significant swelling and disruption of mitochondria, as well as myofibril disarray, in the LV myocardium of the mock group (Fig. 1A). However, these abnormalities in LV ultrastructure were markedly improved by Ang-(1–7) or perindopril treatment. The effects of Ang-(1–7) on LV ultrastructural abnormalities were blocked in part by co-administration of A779 or PD123319.

### Ang-(1–7) suppresses myocardial fibrosis

In the mock group, the collagen volume fraction (CVF) and ratio of perivascular collagen area to luminal area (PVCA/LA) increased by 2.9- and 3.1-fold, respectively, as compared with the control group, and these values were significantly reduced by the Ang-(1–7) treatment at the end of week 16. Moreover, CVF and PVCA/LA were greatly decreased with combined Ang-(1–7) and perindopril treatment relative to single therapy with Ang-(1–7) or perindopril. However, the beneficial effects of Ang-(1–7) on CVF and PVCA/LA were completely reversed by A779 treatment (Fig. 1A–C).

**Figure 2:**
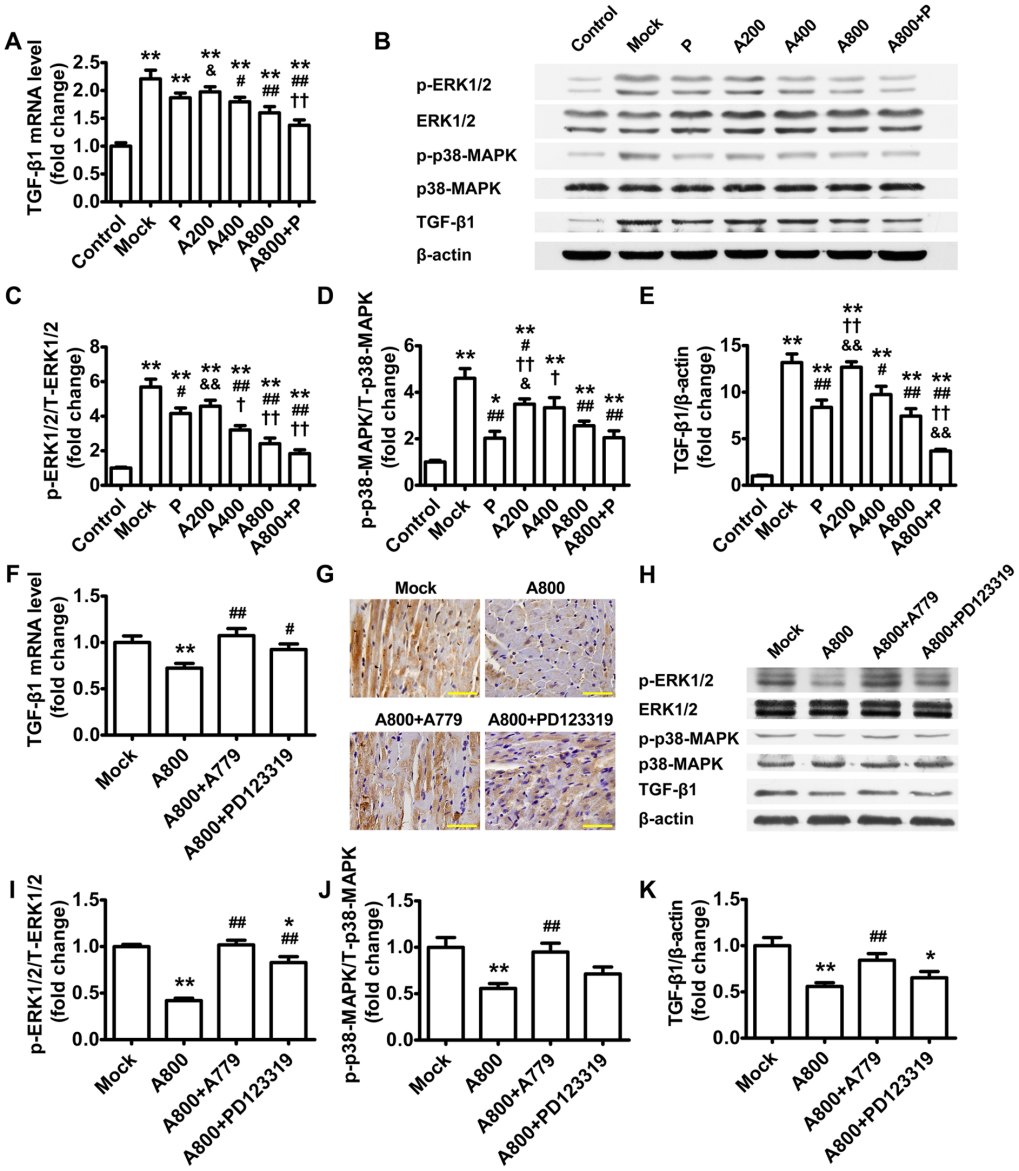
TGF-β1 expression and activation of ERK1/2 and p38-MAPK in rat hearts. Ang-(1–7) treatment suppresses transforming growth factor (TGF)-β1 expression and activation of ERK1/2 and p38-MAPK in diabetic hearts. (A) Relative mRNA expression of myocardial TGF-β1 and (B) representative western blot analysis of phophorylated ERK1/2 (p-ERK1/2), ERK1/2, p-p38-MAPK, p38-MAPK, TGF-β1 and β-actin in 7 groups of rats. Quantification of p-ERK1/2/ERK1/2 (C), p-p38/p38 (D) and TGF-β1 protein expression levels (E). *P < 0.05 and **P < 0.01 vs. control; ^#^*P* < 0.05 and ^##^*P* < 0.01 vs. mock; ^†^*P* < 0.05 and ^††^*P* < 0.01 vs. perindopril (P); ^&^*P* < 0.05 and ^&&^*P* < 0.01 vs. high-dose Ang-(1–7) (800 ng·kg^−1^·min^−1^) (A800). The inhibitory effects of Ang-(1–7) on TGF-β1 expression and ERK1/2 and p38-MAPK activation were completely reversed by co-administration of A779 and partially blocked by PD123319. (F) Relative mRNA expression of myocardial TGF-β1 and (G) representative immunohistochemical staining for TGF-β1 in 4 groups of rats (scale bar: 50 μm). (H) Representative western blot analysis of p-ERK1/2, ERK1/2, p-p38-MAPK, p38-MAPK, TGF-β1 and β-actin in 4 groups of rats. Quantification of p-ERK1/2/ERK1/2 (I), p-p38/p38 (J) and TGF-β1 (K). *P < 0.05 and **P < 0.01 vs. mock; ^#^*P* < 0.05 and ^##^*P* < 0.01 vs. A800. Abbreviations are in Figure 1. Cropped blots are used in Figure 2, and the blots were run under the same experimental conditions.

As compared with the mock group, Ang-(1–7) treatment dose-dependently reduced mRNA expression of fibrosis-associated genes, including fibronectin-1 (Fig. 1D), collagen I-α1 (Fig. 1E) and TGF-β1 (Fig. 2A), as well as the ratio of collagen I-α1 to III-α1 (Fig. 1G). Similar effects were observed in the perindopril group. Again, these Ang-(1–7)–induced effects were completely reversed by A779 and partially blocked by PD123319 (Figs. 1D, E, G, and 2F). Immunohistochemistry revealed that the contents of collagen I and III and the ratio of collagen I to III were significantly higher in the mock than control group (Fig. 1H–K). Compared with the mock group, Ang-(1–7) treatment reduced the content of collagen I and ratio of collagen I to III (Fig. 1H, I, and K) but not content of collagen III (Fig. 1H and J). The effect of Ang-(1–7) on collagen I expression was reversed completely by A779 and partially by PD123319.

In addition, Ang-(1–7) treatment dose-dependently inhibited activation of ERK1/2 and p38-MAPK and TGF-β1 protein expression as compared with mock treatment (Fig. 2B–E). The inhibitory effects of Ang-(1–7) on ERK1/2 and p38-MAPK activation and TGF-β1 expression were completely reversed by A779 and partially by PD123319 (Fig. 2G–K).

### Ang-(1–7) attenuates myocardial hypertrophy

**Figure 3:**
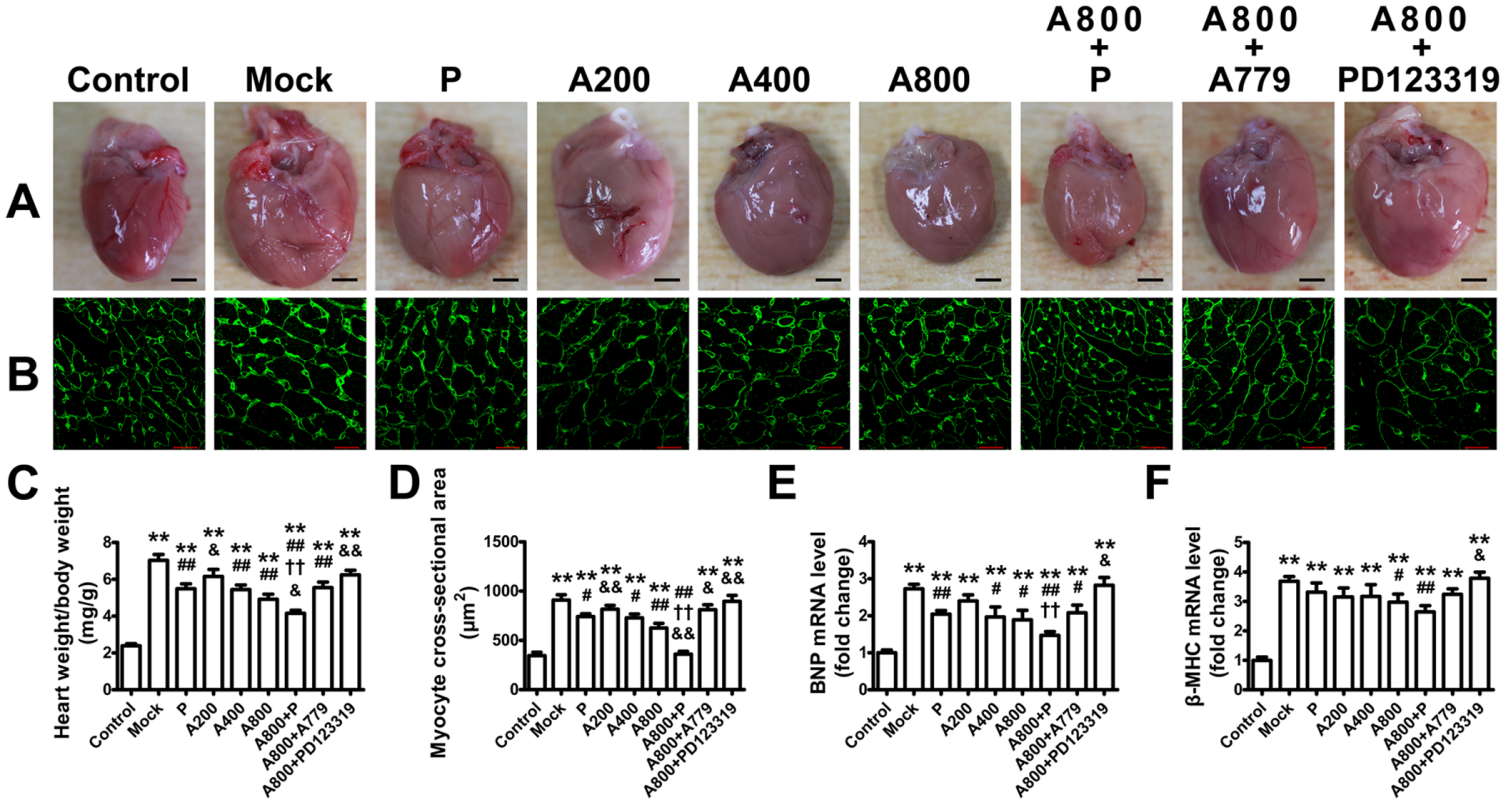
Cardiac hypertrophy in 9 groups of rats. (A) Representative heart size in 9 groups of rats (scale bar: 2 mm). (B) Representative left ventricular sections stained with wheat germ agglutinin (scale bar: 20 μm). Quantitative analysis of the ratio of heart weight to body weight (C), myocyte cross-sectional area (D) and relative mRNA levels of brain natriuretic protein (BNP) (E) and β-myosin heavy chain (β-MHC) (F). **P < 0.01 vs. control; ^#^*P* < 0.05 and ^##^*P* < 0.01 vs. mock; ^††^*P* < 0.01 vs. perindopril (P); ^&^*P* < 0.05 and ^&&^*P* < 0.01 vs. high-dose Ang-(1–7) (800 ng·kg^−1^·min^−1^) (A800). Abbreviations are in Figure 1.

Intraventricular septal thickness (IVSth), LV posterior wall thickness (LVPWth) and ratio of heart weight to body weight were significantly higher in the mock than control group (Table 1 and Fig. 3A and C). Treatment with Ang-(1–7) dose-dependently reduced IVSth, LVPWth and the ratio of heart weight to body weight compared with the mock group. Notably, combined treatment with Ang-(1–7) and perindopril further reduced IVSth, LVPWth and ratio of heart weight to body weight as compared with single treatment. However, the effects of Ang-(1–7) on myocardial hypertrophy were largely reversed by co-administration of PD123319 or A779.

Cardiomyocyte cross-sectional areas were significantly larger in the mock than control group (Fig. 3B and D), which indicates myocardial hypertrophy in DCM rats. In contrast, Ang-(1–7)–treated groups showed a dose-dependent decrease in the cardiomyocyte cross-sectional areas as compared with the mock group, and this effect was enhanced by co-administration of perindopril and reversed by PD123319 or A779. Similarly, the mRNA expression of brain natriuretic peptide and β-myosin heavy chain as markers of cardiac hypertrophy was dose-dependently reduced by Ang-(1–7) treatment, which was also enhanced by co-administration of perindopril and completely reversed by PD123319 (Fig. 3E and F).

### Ang-(1–7) inhibits cardiac apoptosis

**Figure 4:**
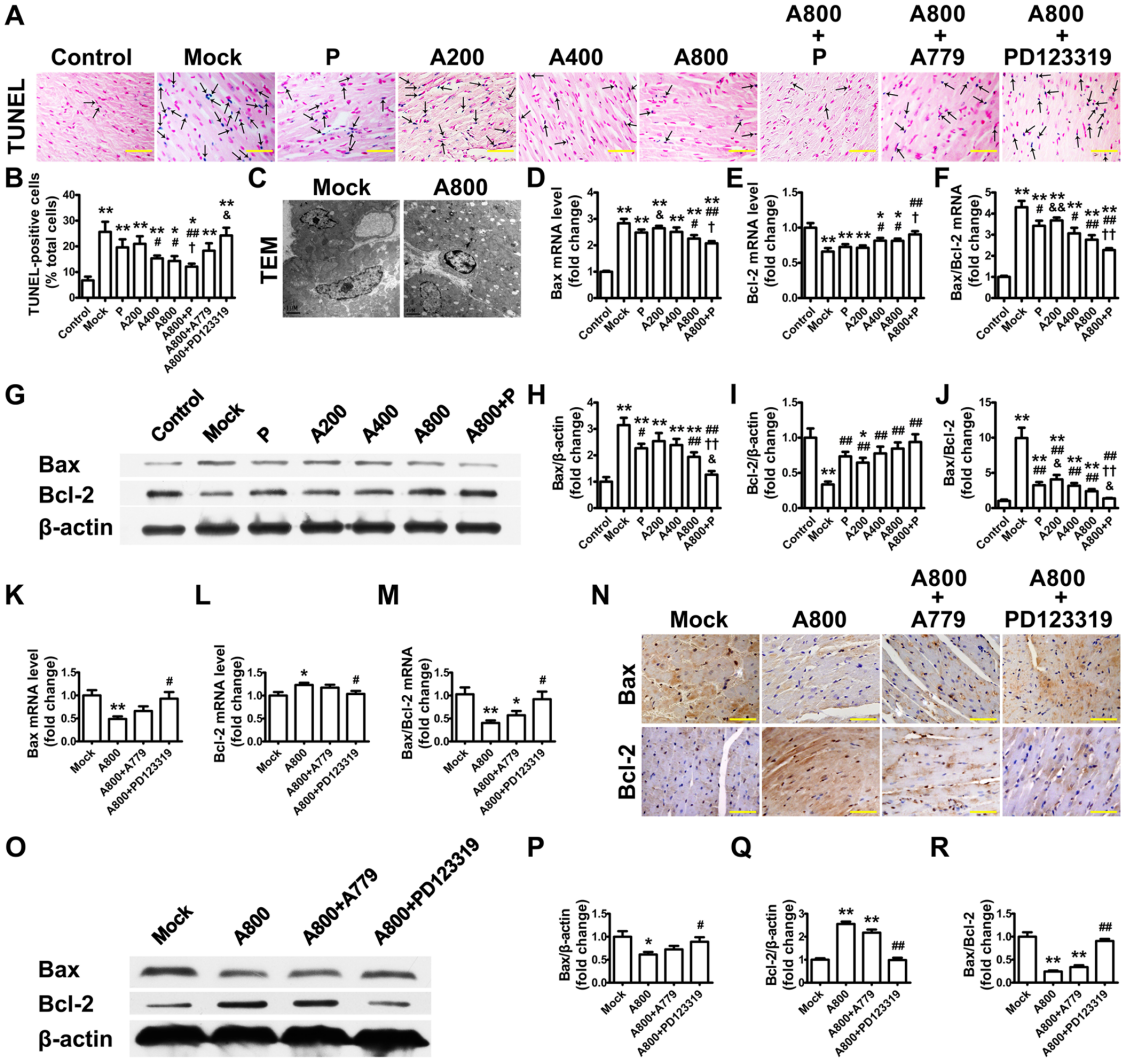
Myocyte apoptosis in rat hearts. (A) Representative TUNEL staining (dark blue; black arrows) for DNA fragmentation in situ with Nuclear Fast Red counterstaining (red) in 9 groups of rats (scale bar: 50 μm). (B) Quantitative analysis of TUNEL-positive staining cells. *P < 0.05 and **P < 0.01 vs. control; ^#^*P* < 0.05 and ^##^*P* < 0.01 vs. mock; ^†^*P* < 0.05 vs. perindopril (P); ^&^*P* < 0.05 vs. high-dose Ang-(1–7) (800 ng·kg^−1^·min^−1^) (A800). (C) Representative TEM of mock group showing nuclei of 2 cardiac myocytes with irregular nuclear outline and margination of their chromatin (left) and normalization of nuclei after Ang-(1–7) treatment at 800 ng·kg^−1^·min^−1^ (A800) (right, scale bar: 1 μm). Relative mRNA expression of myocardial Bax (D), Bcl-2 (E) and ratio of Bax to Bcl-2 (F) and (G) representative western blot analysis of protein expression levels of Bax, Bcl-2, and β-actin in 7 groups of rats. Quantification of Bax (H) and Bcl-2 (I) expression and the ratio of Bax to Bcl-2 (J). *P < 0.05 and **P < 0.01 vs. control; ^#^*P* < 0.05 and ^##^*P* < 0.01 vs. mock; ^†^*P* < 0.05 and ^††^*P* < 0.01 vs. P; ^&^*P* < 0.05 and ^&&^*P* < 0.01 vs. A800. The effects of Ang-(1–7) on Bax and Bcl-2 expression and the ratio of Bax to Bcl-2 were completely reversed by PD123319 and partially blocked by A779. Relative mRNA expression of myocardial Bax (K), Bcl-2 (L) and the ratio of Bax to Bcl-2 (M) in 4 groups of rats. (N) Representative immunohistochemical staining for Bax (top panel) and Bcl-2 (bottom panel) (scale bar: 50 μm). (O) Representative western blot analysis of Bax, Bcl-2 and β-actin. Quantification of Bax (P), Bcl-2 (Q) and ratio of Bax to Bcl-2 (R). *P < 0.05 and **P < 0.01 vs. mock; ^#^*P* < 0.05 and ^##^*P* < 0.01 vs. A800. Abbreviations are in Figure 1. Cropped blots are used in Figure 4, and the blots were run under the same experimental conditions.

Rats in the mock group showed prominent cardiac apoptosis, as indicated by significantly increased proportion of TUNEL-positive cells (Fig. 4A and B), abnormal morphology of myocyte nuclei by TEM (Fig. 4C), increased mRNA and protein expression of Bax and ratio of Bax to Bcl-2 (Fig. 4D, F–H, and J) and significantly decreased mRNA and protein expression of Bcl-2 (Fig. 4E, G, and I), all of which were ameliorated by treatment with Ang-(1–7) at 800 ng·kg^−1^·min^−1^. Of note, Ang-(1–7) at 800 ng·kg^−1^·min^−1^ improved features of cardiomyocyte nuclei (Fig. 4C). The effects of Ang-(1–7) on cardiac apoptosis were completely reversed by PD123319 and partially blocked by A779 (Fig. 4A, B, and K–R).

### Ang-(1–7) ameliorates myocardial oxidative stress and inflammation

Consistent with a previous report that oxidative stress may be a pivotal mechanism of high glucose-mediated cardiovascular injury^16^, we found greater O_2_^−^ production and NADPH oxidase activation in myocardia of the mock than control group. Ang-(1–7) treatment dose-dependently attenuated and the combined Ang-(1–7) and perindopril normalized O_2_^–^ production and NADPH oxidase activation (Supplementary Fig. 2A and B). These effects were offset by co-administration of A779 or PD123319.

On immunohistochemical staining of myocardia, the protein expression of IL-1β, IL-6 and MCP-1 was greater in the mock than control group (Supplementary Fig. 2A and C). Ang-(1–7) treatment dose-dependently attenuated and combined treatment of Ang-(1–7) and perindopril normalized the expression of IL-1β, IL-6 and MCP-1. These inhibitory effects were reversed by co-administration with A779 or PD123319.

### Ang-(1–7) reduces collagen synthesis of cardiac fibroblasts

High-glucose stimulation significantly increased types I and III collagen content in the cultured media of cardiac fibroblasts (Supplementary Fig. 3A and B), which was inhibited by Ang-(1–7) treatment time- and dose-dependently (Supplementary Fig. 3A–D) as was the ratio of collagen I to III (Supplementary Fig. 3G). These inhibitory effects of Ang-(1–7) were enhanced by combined treatment with perindopril and completely reversed by A779 (Supplementary Fig. 3E–G).

High glucose induced a 2.2-fold increase in collagen synthesis, which was significantly reduced by 10^−5^ M Ang-(1–7) over 72 hr and further decreased by Ang-(1–7) combined with perindopril treatment (Supplementary Fig. 3H). The effect of Ang-(1–7) on collagen synthesis was completely blocked by A779.

### Ang-(1–7) inhibits proliferation, differentiation and oxidative stress of cardiac fibroblasts

**Figure 5:**
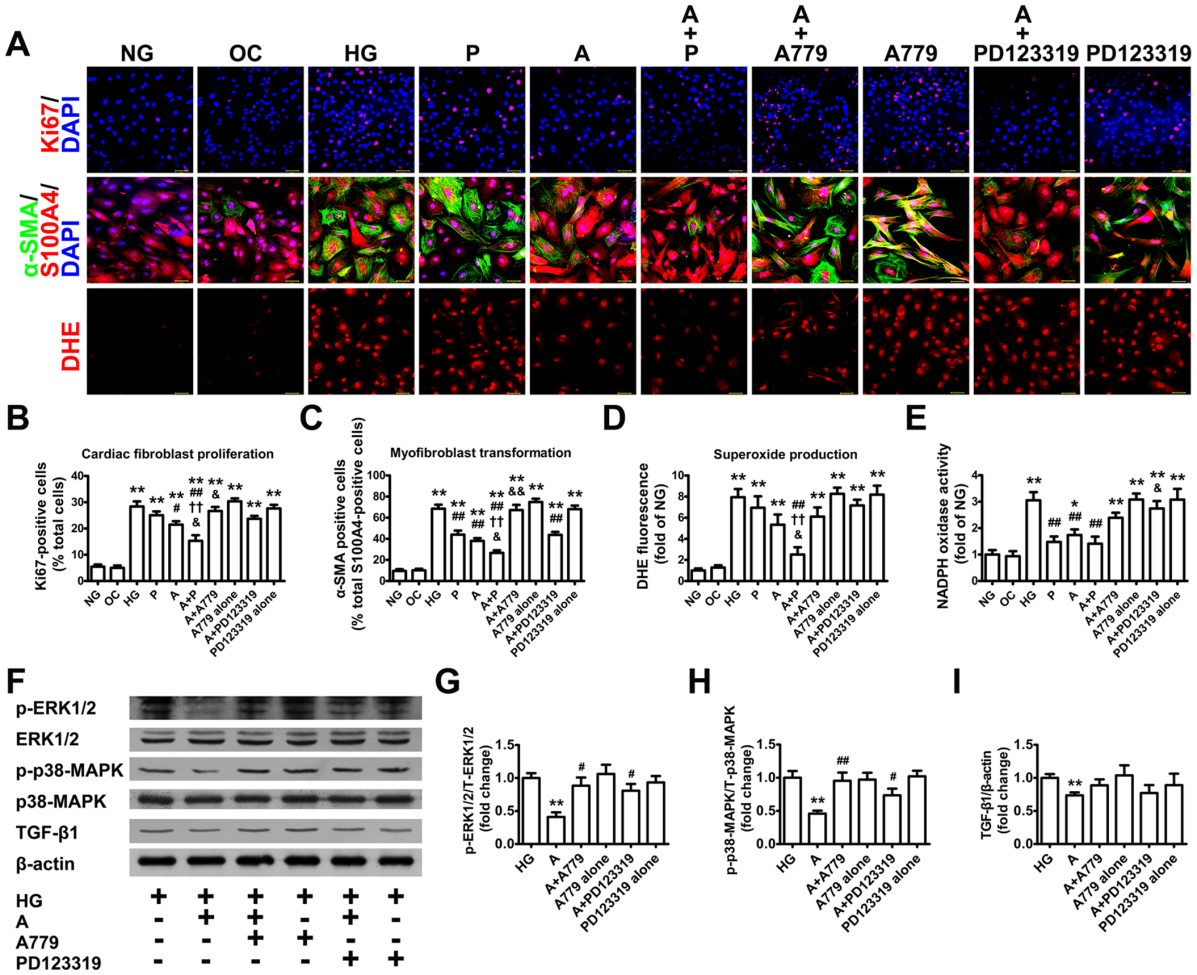
Cell proliferation and differentiation, oxidative stress, ERK1/2 and p38-MAPK phosphorylation and TGF-β1 expression in cultured cardiac fibroblasts. (A) Top panel: cardiac fibroblast proliferation by Ki67 staining (red: nuclei) and DAPI counterstaining (blue: nuclei) (scale bar: 50 μm). Mid panel: myofibroblast transformation by α-smooth muscle actin (α-SMA) staining (green) with counterstaining for S100A4 (red) and nuclei (DAPI; blue) in cardiac fibroblasts (scale bar: 50 μm). Bottom panel: representative dihydroethidium (DHE) fluorescence images of cardiac fibroblasts (scale bar: 50 μm). (B) Quantification of Ki67-positive cells. (C) Quantification of α-SMA–positive cells as a percentage of total S100A4-positive cells. Relative fluorescence DHE values (D) and NADPH oxidase activity (E) in cardiac fibroblasts. *P < 0.05 and **P < 0.01 vs. normal glucose (NG); ^#^*P* < 0.05 and ^##^*P* < 0.01 vs. high glucose (HG); ^††^*P* < 0.01 vs. HG + perindopril (P); ^&^*P* < 0.05 and ^&&^*P* < 0.01 vs. HG + Ang-(1–7) (A). (F) Representative western blot analysis of phophorylated ERK1/2 (p-ERK1/2), ERK1/2, p-p38-MAPK, p38-MAPK, TGF-β1 and β-actin and quantification of levels of p-ERK1/2/ERK1/2 (G) and p-p38/p38 (H) and TGF-β1 (I). **P < 0.01 vs. HG; ^#^*P* < 0.05 and ^##^*P* < 0.01 vs. A. OC = osmotic control. Cropped blots are used in Figure 5, and the blots were run under the same experimental conditions.

High glucose stimulation significantly augmented fibroblast proliferation and differentiation into myofibroblasts (Fig. 5A–C). Ang-(1–7) significantly decreased fibroblast proliferation and differentiation over 72-hr culture versus high-glucose treatment alone; this effect of Ang-(1–7) was completely reversed by A779. Moreover, combined treatment with Ang-(1–7) and perindopril significantly inhibited fibroblast proliferation and differentiation relative to Ang-(1–7) or perindopril alone.

O_2_^−^ level and NADPH oxidase activity were increased by high-glucose stimulation in cultured cardiac fibroblasts; the upregulated NADPH oxidase activation was attenuated by Ang-(1–7) for 72 hr (Fig. 5A, D, and E). Combined incubation with Ang-(1–7) and perindopril normalized the O_2_^−^ level and NADPH oxidase activity in cardiac fibroblasts. In addition, the ability of Ang-(1–7) to attenuate oxidative stress of cardiac fibroblasts induced by high glucose was partially blunted by co-administration of A779 or PD123319.

### Ang-(1–7) inhibits ERK1/2 and p38-MAPK phosphorylation and TGF-β1 expression in cardiac fibroblasts

The ratio of phosphorylated to total protein expression of ERK1/2 and p38-MAPK in cardiac fibroblasts was markedly lower after Ang-(1–7) treatment than high-glucose treatment (Fig. 5F–H). Likewise, the protein expression level of TGF-β1 in cardiac fibroblasts was lower with Ang-(1–7) and high glucose than with high glucose alone (Fig. 5F and I).

The effects of Ang-(1–7) on ERK1/2, p38-MAPK and TGF-β1 in cardiac fibroblasts incubated with high glucose were inhibited by co-administration of A779 or PD123319, so Ang-(1–7) inhibited ERK1/2 and p38-MAPK activation and TGF-β1 expression by binding to both MasR and AT_2_R.

### Ang-(1–7) suppresses fibroblast–myocyte communication

The protein expression of collagen I and III and TGF-β1 was significantly higher in fibroblasts + non-treated myocytes than fibroblasts alone. In contrast, the protein expression of collagen I and III and TGF-β1 were lower in fibroblasts + Ang-(1–7)–treated myocytes than fibroblasts + non-treated myocytes (Supplementary Fig. 4A–C).

The protein expression of collagen I and III and TGF-β1 was substantially higher in fibroblasts + non-treated myocytes than fibroblasts alone. In contrast, the protein levels of collagen I and III and TGF-β1 were lower in fibroblasts + Ang-(1–7)-treated myocytes than fibroblasts + non-treated myocytes (Supplementary Fig. 4D–F).

The inhibitory effects of Ang-(1–7) on collagen and TGF-β1 production induced by fibroblast–myocyte communication were partially blocked by co-administration of A779 or PD123319.

### Ang-(1–7) prevents cardiomyocyte hypertrophy, apoptosis and oxidative stress via MasR and AT_2_R

In *in vitro* experiments, high glucose significantly increased cardiomyocyte size and apoptosis rate as compared with normal glucose (Supplementary Fig. 5A, B and C). Ang-(1–7) treatment normalized cardiomyocyte size and apoptosis rate as compared with high-glucose treatment alone, which were abrogated by co-treatment with A779 or PD123319. Neither A779 nor PD123319 alone altered the size and apoptosis rate of high-glucose–incubated cardiomyocytes.

O_2_^−^ level and NADPH oxidase activity were higher with high glucose than normal-glucose treatment (Supplementary Fig. 5D and E). Ang-(1–7) treatment with or without administration of perindopril normalized high glucose-induced O_2_^−^ production and NADPH oxidase activation in cardiac myocytes. In addition, the ability of Ang-(1–7) to attenuate oxidative stress of cardiac myocytes after high-glucose stimulation was partially blocked by co-administration of A779 or PD123319.

### Ang-(1–7) downregulates AT_1_R and upregulates AT_2_R *in vivo* and *in vitro*

**Figure 6:**
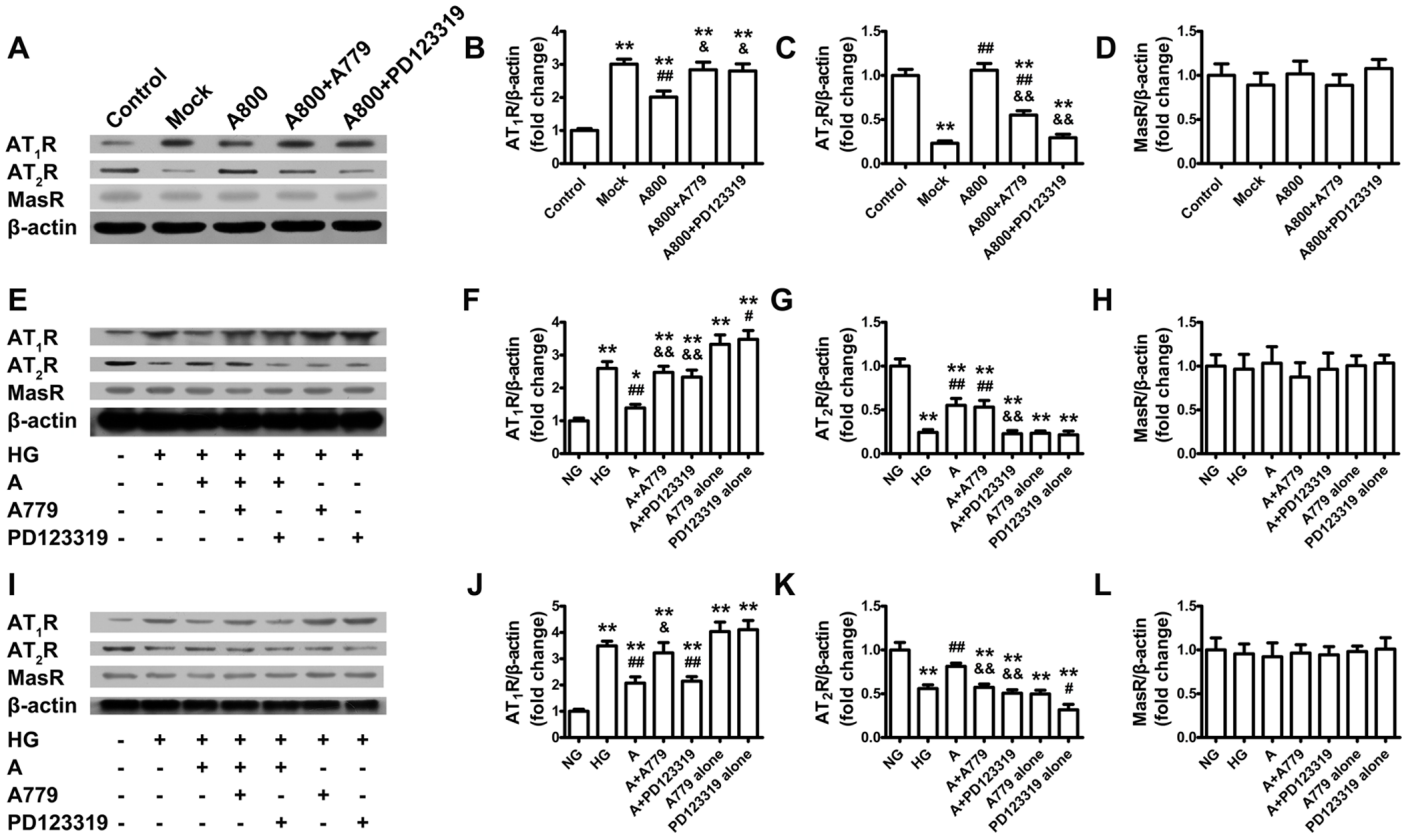
AT_1_R, AT_2_R and MasR protein expression *in vivo* and *in vitro*. (A) Representative western blot analysis of Ang-II type 1 receptor (AT_1_R), Ang-II type 2 receptor (AT_2_R), Mas receptor (MasR) and β-actin protein expression in 5 groups of rats. Quantification of AT_1_R (B), AT_2_R (C) and MasR (D) protein expression. **P < 0.01 vs. control; ^##^*P* < 0.01 vs. mock; ^&^*P* < 0.05 and ^&&^*P* < 0.01 vs. high-dose Ang-(1–7) (800 ng·kg^−1^·min^−1^) (A800). (E) Representative western blot analysis of AT_1_R, AT_2_R, MasR and β-actin protein expression in cardiofibroblasts. Quantification of AT_1_R (F), AT_2_R (G) and MasR (H) protein expression. *P < 0.05 and **P < 0.01 vs. normal glucose (NG); ^#^*P* < 0.05 and ^##^*P* < 0.01 vs. high glucose (HG); ^&&^*P* < 0.01 vs. HG + Ang-(1–7) (A). (I) Representative western blot analysis of AT_1_R, AT_2_R, MasR and β-actin protein expression in cardiomyocytes. Quantification of AT_1_R (J), AT_2_R (K) and MasR (L) protein expression. **P < 0.01 vs. NG; ^#^*P* < 0.05 and ^##^*P* < 0.01 vs. HG; ^&^*P* < 0.05 and ^&&^*P* < 0.01 vs. A. Cropped blots are used in Figure 6, and the blots were run under the same experimental conditions.

*In vivo*, AT_1_R expression was significantly increased and AT_2_R expression was decreased with mock than control treatment. These effects were largely attenuated by high-dose Ang-(1–7) (Fig. 6A–C). *In vitro*, high glucose induced higher AT_1_R expression and lower AT_2_R expression than normal glucose in both cardiac fibroblasts and myocytes, which was reversed by Ang-(1–7) treatment (Fig. 6E–G and I–K). Nevertheless, Ang-(1–7) did not affect MasR expression in rat hearts (Fig. 6A and D) or cardiac fibroblasts (Fig. 6E and H) and myocytes (Fig. 6I and L) cultured in high-glucose medium.

The effect of Ang-(1–7) on AT_1_R expression in rat hearts and cultured cardiac fibroblasts was completely inhibited with co-administration of A779 or PD123319, and that in cardiac myocytes was reversed by A779 but not by PD123319. The effect of Ang-(1–7) on AT_2_R expression in rat hearts was completely reversed by PD123319 and partially by A779; that in cardiac fibroblasts was completely blocked by PD123319 but not altered by A779; and that in cardiac myocytes was totally inhibited by A779 or PD123319.

### Ang-(1–7) increases Ang-(1–9) levels and ACE2 activity

At the end of week 12, the plasma Ang-II level was higher in the model than control group, with no significant differences in plasma Ang-(1–7) and Ang-(1–9) levels between normal and diabetic rats (Supplementary Table 6). Both plasma (Supplementary Table 7) and myocardial (Supplementary Table 8) levels of Ang-(1–7) were significantly increased after 4-week treatment with Ang-(1–7) and further increased by Ang-(1–7) with perindopril, which suggests that exogenous Ang-(1–7) was taken up by the myocardium through circulation and perindopril prevented the catalytic metabolism of Ang-(1–7) into inactive Ang-(1–5). Moreover, both plasma (Supplementary Table 7) and myocardial (Supplementary Table 8) Ang-(1–9) levels were significantly increased by high-dose Ang-(1–7) or perindopril treatment. However, Ang-(1–7) did not affect Ang-II levels in the plasma (Supplementary Table 7) and the myocardium (Supplementary Table 8) *in vivo*, nor in the cardiac fibroblasts (Supplementary Fig. 6A) and myocytes (Supplementary Fig. 6B) cultured in high-glucose medium. Ang-(1–7) treatment did not alter myocardial ACE activity (Supplementary Fig. 7A) or plasma ADAM17 activity (Supplementary Fig. 8A) but significantly increased ACE2 activity in both the myocardium and plasma (Supplementary Figs. 7B and 8B). Furthermore, Ang-(1–7)-mediated upregulation of Ang-(1–9) level and ACE2 activity was blocked by PD123319 administration.

## Discussion

The major finding of the present study was that chronic Ang-(1–7) treatment protected against LV remodeling and dysfunction in DCM rats without altering body weight, blood pressure, heart rate or blood glucose level. The cellular mechanisms may involve decreased fibroblast proliferation and differentiation into myofibroblasts, attenuated mitochondria swelling, myofibril disarray, hypertrophy and apoptosis of cardiomyocytes, as well as inhibited fibroblast–myocyte communication. The molecular mechanisms may involve reduced inflammatory cytokine expression, oxidative stress and collagen synthesis, inhibited ERK1/2 and p38-MAPK phosphorylation and TGF-β1 expression, upregulated ACE2 activity and Ang-(1–9) level, as well as a complex interaction of MasR, AT_2_R and AT_1_R (Supplementary Fig. 9). Furthermore, Ang-(1–7) combined with perindopril provided additional cardioprotection, a finding of important clinical implications in the development of a novel therapeutic regimen for DCM.

DCM is characterized by cardiac fibrosis, myocardial hypertrophy and myocyte apoptosis; the most salient pathological feature of DCM is the accumulation of collagen in the extracellular matrix. A wealth of evidence has confirmed the importance of RAS in collagen production in DCM. Therefore, most recent studies have focused on discovering novel therapeutic targets from RAS^3^. Although ACE inhibitors or AT_1_R antagonists have been found effective in preventing diabetic complications, recent studies revealed that AT_1_R antagonists can inhibit the effects of only extracellular Ang-II^3^; however, intracellular Ang-II is an important mediator of collagen production in DCM. ACE inhibitors can block Ang-II synthesis catalyzed by ACE in cardiac fibroblasts but not that catalyzed by chymase in cardiac myocytes^3^,^17^. Thus, ACE inhibitors and AT_1_R antagonists can only partially inhibit RAS activities in DCM. In contrast, Ang-(1–7) has powerful anti-oxidative stress effects opposite to those of Ang-II in both fibroblasts and myocytes.

In the pathological process of myocardial fibrosis, cardiac fibroblasts play an important role by differentiating into myofibroblasts, which may induce fibroblast proliferation. Myofibroblasts, which are absent in the normal myocardium, are characterized by acquisition of α-smooth muscle actin expression and enhanced collagen synthesis and constitute the major cellular source of collagen production in myocardial fibrosis^18^. Thus, inhibition of the differentiation of cardiac fibroblasts to myofibroblasts is considered a key target for anti-fibrosis therapy. In addition, recent studies found that after high glucose or Ang-II stimulation, myocytes may secrete active TGF-β that may induce collagen synthesis in fibroblasts^5^,^19^, which suggests that the cross-talk between myocytes and fibroblasts may play an important role in the pathogenesis of DCM. Inhibition of the cross-talk between myocytes and fibroblasts is an indispensable mechanism underlying the therapeutic effects of Ang-(1–7) in DCM.

The molecular mechanisms of cardiac fibrosis are complex and involve a cascade of intracellular signaling pathways. TGF-β1 is a key pro-fibrotic cytokine markedly elevated in experimental DCM. The finding that Ang-(1–7) inhibited the expression of TGF-β1 in this study indicates that suppression of the TGF-β1 pathway underlies the anti-fibrotic effects of Ang-(1–7) in DCM. Previous studies implicated ERK1/2 and p38-MAPK pathways in cardiac fibrosis and hypertrophy^20^,^21^ and our study demonstrated that Ang-(1–7) significantly inhibited both phosphorylation of ERK1/2 and p38-MAPK in diabetic hearts and high glucose-induced activation of ERK1/2 and p38-MAPK in cardiac fibroblasts, which agreed with recent reports in other disease models^22^,^23^,^24^. Recently, emerging evidence suggests that the effects of Ang-(1–7) may be mediated by activation of some other phosphatases including Src homology 2-containing protein-tyrosine phosphatase-1, phosphatase and tensin homologue, and dual-specificity phosphatase 1^22^,^23^,^25^.

Whether inflammation plays a role in the pathogenesis of DCM remains disputed. Accumulating evidence suggests that increased oxidative stress coupled with activation of downstream pro-inflammatory pathways leads to LV remodeling and dysfunction in DCM^2^, and other evidence suggests that DCM is different from other types of cardiomyopathy in the lack of an inflammatory response^26^. In the current study, we found that enhanced O_2_^−^ generation, NADPH oxidase activation and proinflammatory cytokine expression in the mock group was inhibited by Ang-(1–7) treatment. Therefore, oxidative stress and inflammation may take part in the pathogenesis of DCM, which was significantly attenuated by Ang-(1–7) treatment.

In the present study, we examined the impact of Ang-(1–7) on the expression of AT_1_R, AT_2_R and MasR. Previous studies showed that upregulated AT_1_R and downregulated AT_2_R promoted cardiac fibrosis and hypertrophy^27^,^28^. We found that AT_1_R expression was significantly increased and AT_2_R expression was decreased in the mock group; these effects were largely reversed by Ang-(1–7) treatment. The mechanism underlying the effects of Ang-(1–7) on AT_1_R is unclear but may involve the complex interactions among AT_1_R, AT_2_R and MasR, because Ang-(1–7) had no effect on Ang-II levels and the effect of Ang-(1–7) on AT_1_R expression was inhibited by A779 or PD123319 *in vivo* and *in vitro*. The effects of Ang-(1–7) on cardiac fibrosis were completely reversed by A779 and partially blocked by PD123319, whereas those on myocardial hypertrophy and myocyte apoptosis were completely reversed by PD123319 and partially blocked by A779. Similar to Ang-II, with two receptors, Ang-(1–7) may exert its cardioprotective effects via both MasR and AT_2_R, and different receptor subtypes may mediate different biological effects. Although Ang-(1–7) appears to act via the MasR *in vitro*^29^, this selectivity is lost *in vivo*^30^. One recent study showed that Ang-(1–7) may act via both AT_2_R and MasR because both PD123319 and A779 abrogated Ang-(1–7)–induced vasoprotection^31^, which lends support to our results.

We found that combining exogenous Ang-(1–7) with perindopril significantly increased plasma and myocardial levels of Ang-(1–7) and provided more cardioprotection than single therapy, probably because perindopril reduces Ang-II generation and inhibits conversion of Ang-(1–7) into inactive Ang-(1–5)^13^. A recent study demonstrated that long-term AT_2_R activation increased renal ACE2 activity^32^. Our results showed that exogenous Ang-(1–7) treatment significantly increased myocardial ACE2 activity and Ang-(1–9) level, possibly via its effect on AT_2_R, and the activated ACE2 may catalyze Ang-II into Ang-(1–7), thus forming a positive feedback. Ang-II was recently found to induce ACE2 shedding by promoting ADAM17 activity as a positive feedback mechanism whereby Ang-II facilitates the loss of its negative regulator, ACE2^33^. Thus, the discrepancy between the increased myocardial ACE2 activity and decreased plasma ACE2 activity with perindopril treatment might be attributed to the reduced ACE2 shedding due to downregulated ADAM17 activity after inhibited generation of Ang-II. However, Ang-(1–7) had no effect on myocardial ACE activity and Ang-II level and plasma ADAM17 activity, suggestive of a negative impact of Ang-(1–7) on ACE2 shedding. Therefore, increased ACE2 activity by Ang-(1–7) and decreased ACE2 shedding by ACE inhibition might offer another advantage of combined therapy versus single therapy.

Several clinical studies reported that elevated plasma ACE2 activity as a compensatory response was associated with increased severity of myocardial dysfunction and was an independent predictor of adverse clinical events^34^,^35^,^36^. Moreover, we recently found that plasma Ang-(1–7) level was independently associated with LV remodeling and dysfunction in diabetic patients^37^ and could also predict myocardial salvage after reperfusion treatment for acute myocardial infarction^38^. Therefore, plasma Ang-(1–7) may be a biomarker for LV function and a predictor for long-term myocardial remodeling under disease conditions. Whether the association of Ang-(1–7) and LV function is attributed to the inhibitory effect of Ang-(1–7) on LV remodeling merits future study.

### Study limitations

The STZ-induced diabetic model has important limitations and our results need to be validated in genetically manipulated diabetic models. Also, although we found that Ang-(1–7) treatment was associated with downregulated AT_1_R, upregulated AT_2_R, attenuated oxidative stress and inflammation, and inhibited fibroblast–myocyte communication, the key mechanism mediating these effects remains obscure and require further investigation. Future research should also focus on the relative AT_1_R-, AT_2_R- and MasR-binding affinities of Ang-(1–7) in the heart.

## Conclusions

Chronic Ang-(1–7) treatment ameliorated LV remodeling and dysfunction in DCM via multiple mechanisms involving attenuated inflammation, oxidative stress and collagen synthesis, inhibited ERK1/2 and p38-MAPK signaling and TGF-β1 expression, upregulated ACE2 activity and Ang-(1–9) level as well as complex interactions of MasR, AT_2_R and AT_1_R. Furthermore, combining Ang-(1–7) with perindopril provided additional cardioprotection. Thus, Ang-(1–7) administration may be a novel and promising approach to the treatment of DCM.

## Supplementary Material

Supplementary Information Combined supplementary information

## Figures and Tables

**Table 1:** Echocardiographic assessment of cardiac structure and function after 4-week treatment

	Control	Mock	P	A200	A400	A800	A800 + P	A800 + A779	A800 + PD123319
IVSth (mm)	1.52 ± 0.10	2.20 ± 0.19*	1.88 ± 0.07*	1.93 ± 0.10*	1.83 ± 0.11	1.80 ± 0.09	1.63 ± 0.11#	1.96 ± 0.14*	2.09 ± 0.25*
LVPWth (mm)	1.51 ± 0.08	2.34 ± 0.20*	1.92 ± 0.11*	1.98 ± 0.07*&	1.90 ± 0.09*	1.74 ± 0.08#	1.50 ± 0.06#†&	1.98 ± 0.15*	2.21 ± 0.26*
LVESD (mm)	2.98 ± 0.05	4.98 ± 0.11*	4.18 ± 0.17*#	3.93 ± 0.15*#	3.76 ± 0.13*#	3.77 ± 0.11*#	3.35 ± 0.16*#†&	4.83 ± 0.06*&	4.25 ± 0.13*#&
LVEDD (mm)	4.95 ± 0.07	7.17 ± 0.13*	6.46 ± 0.19*#	6.06 ± 0.24*#	5.91 ± 0.19*#	5.91 ± 0.14*#†	5.43 ± 0.20*#†	7.14 ± 0.11*&	6.44 ± 0.12*#&
LVEF (%)	70.28 ± 0.92	56.32 ± 2.06*	62.93 ± 2.98*	63.06 ± 2.06*#	64.88 ± 2.57#	64.74 ± 2.06*#	67.64 ± 2.33#	59.07 ± 1.12*&	61.64 ± 2.03*
FS (%)	39.91 ± 0.77	30.50 ± 1.39*	35.17 ± 2.19	34.93 ± 1.53*	36.31 ± 1.95#	36.15 ± 1.56*#	38.20 ± 1.85#	32.33 ± 0.80*	34.02 ± 1.48*
E/A ratio	2.07 ± 0.08	1.10 ± 0.07*	1.27 ± 0.07*	1.23 ± 0.09*	1.43 ± 0.08*#	1.53 ± 0.11*#	1.83 ± 0.04*#†&	1.18 ± 0.06*&	1.33 ± 0.10*
E′/A′ ratio	1.57 ± 0.04	0.71 ± 0.04*	0.97 ± 0.06*#	0.95 ± 0.07*#	1.14 ± 0.07*#	1.18 ± 0.10*#	1.27 ± 0.08*#†	0.88 ± 0.07*#&	1.01 ± 0.07*#

*P < 0.05 vs. control. ^#^*P* < 0.05 vs. mock. ^†^*P* < 0.05 vs. perindopril (P). ^&^*P* < 0.05 vs. high-dose Ang-(1–7) (800 ng·kg^−1^·min^−1^) (A800).

A200 = Ang-(1–7) at 200 ng·kg^−1^·min^−1^; A400 = Ang-(1–7) at 400 ng·kg^−1^·min^−1^; E/A ratio = ratio of early to late left ventricular filling velocity; E'/A' ratio = ratio of early to late diastolic peak annular velocity; FS = fraction shortening; IVSth = intraventricular septal thickness; LVEDD = left ventricular end-diastolic diameter; LVEF = left ventricular ejection fraction; LVESD = left ventricular end-systolic diameter; LVPWth = left ventricular posterior wall thickness.

**Table 2:** Hemodynamic assessment of cardiac function after 4-week treatment

	Control	Mock	P	A200	A400	A800	A800 + P	A800 + A779	A800 + PD123319
LVSP (mmHg)	126.5 ± 2.8	97.9 ± 3.5*	107.6 ± 2.9*	107.8 ± 3.0*	112.7 ± 3.2*#	116.8 ± 3.0*#†	122.0 ± 2.8#†	106.2 ± 2.7*&	111.8 ± 3.1*#
LVEDP (mmHg)	5.63 ± 0.38	15.75 ± 0.82*	11.29 ± 0.42*#	13.67 ± 0.61*†&	11.33 ± 0.49*#	10.17 ± 0.60*#	8.83 ± 0.48*#†	14.50 ± 1.18*&	12.83 ± 0.65*#&
+dp/dt (mmHg/sec)	7112 ± 258	4450 ± 90*	5876 ± 74*#	5210 ± 86*#†&	5853 ± 97*#	6042 ± 78*#	6335 ± 169*#†	5108 ± 121*#&	5609 ± 157*#&
−dp/dt (mmHg/sec)	5145 ± 44	4167 ± 50*	4584 ± 64*#	4528 ± 101*#	4696 ± 88*#	4690 ± 87*#	5021 ± 109#†&	4304 ± 99*&	4549 ± 70*#

*P < 0.05 vs. control. ^#^*P* < 0.05 vs. mock. ^†^*P* < 0.05 vs. perindopril (P). ^&^*P* < 0.05 vs. high-dose Ang-(1–7) (800 ng·kg^−1^·min^−1^) (A800).

+dp/dt = maximal rate of pressure rise; −dp/dt = maximal rate of pressure fall; LVEDP = left ventricular end-diastolic pressure; LVSP = left ventricular systolic pressure; other abbreviations are in Table 1.
