# Supplementary material for: Combination of angiotensin-(1–7) with perindopril is better than single therapy in ameliorating diabetic cardiomyopathy
Source: Sci Rep. 2015 Mar 5;5:8794. doi: 10.1038/srep08794 (PMC4350094; doi:10.1038/srep08794)

**Combination of angiotensin-(1–7) with perindopril is better than single therapy in ameliorating diabetic cardiomyopathy**

***Running head:*** Angiotensin-(1–7) mitigates diabetic cardiomyopathy

***By:***

Panpan Hao,^1^* Jianmin Yang,^1^* Yanping Liu,^2^ Mingxiang Zhang,^1^ Kai Zhang,^1^ Fei Gao,^1^ Yuguo Chen,^1^ Cheng Zhang,^1^ Yun Zhang^1^

***From:***

^1^The Key Laboratory of Cardiovascular Remodeling and Function Research, Chinese Ministry of Education and Chinese Ministry of Public Health, Qilu Hospital, Shandong University, Jinan 250012, Shandong, China

^2^Shandong Provincial Key Laboratory of Diagnosis and Treatment of Cardio-cerebral Vascular Diseases, Shandong Medical Imaging Research Institute, Shandong University, Jinan 250021, S handong, China

**Correspondence and requests for materials should be addressed to** C.Z. ([zhangc@sdu.edu.cn](mailto:zhangc@sdu.edu.cn)) or Y.Z. ([zhangyun@sdu.edu.cn](mailto:zhangyun@sdu.edu.cn))

* These authors contributed equally to this work.

**Supplementary materials and methods**

**Animal model.** We purchased 126 male Wistar rats (approximately 200g in weight) from Shandong University Animal Center and housed them in temperature- (22 ± 2 °C) and humidity-controlled (55 ± 5%) rooms on a 12-h light/12-h dark cycle. During the experimental period, food and water were provided ad libitum, and animals were weighed initially and then biweekly throughout the experiment. After one week of acclimatization, rats were divided randomly into 2 groups: model (n = 112) and control (n = 14). Diabetes was induced in the model group by a single intraperitoneal injection of streptozotocin (Sigma, St. Louis, MO) at 65 mg· kg^−1^ dissolved in 0.1 M citrate buffer (pH 4.5)^1^. The development of diabetes was confirmed 1 week later by a blood glucose level > 16.7 mM in two consecutive measurements. The control group received an intraperitoneal injection of citrate buffer alone. At the end of week 12 after injection, all rats in the model group were randomly divided into 8 groups for treatment: mock, vehicle treatment only; perindopril, oral perindopril, 2 mg·kg^-1^·d^-1^ (Servier, Melbourne, Australia); low-dose angiotensin-(1–7) [Ang-(1–7)], subcutaneous administration of Ang-(1–7), 200 ng·kg^-1^·min^-1^ (Auspep, Parkville, Australia); moderate-dose Ang-(1–7), subcutaneous administration of Ang-(1–7), 400 ng·kg^-1^·min^-1^; high-dose Ang-(1–7), subcutaneous administration of Ang-(1–7), 800 ng·kg^-1^·min^-1^; high-dose Ang-(1–7) + perindopril; high-dose Ang-(1–7) + A779, a Mas receptor (MasR) antagonist, 800 ng·kg^-1^·min^-1^ (Auspep, Parkville, Australia); and high-dose Ang-(1–7) + PD123319, an angiotensin II type 2 receptor (AT_2_R) antagonist, 10 mg·kg^-1^·day^-1^ (Pfizer, Groton, CT). After anesthesia with an intraperitoneal injection of pentobarbital (60 mg·kg^-1^), Alzet osmotic pumps (Alza Corp, Palo Alto, CA) were implanted for continuous delivery of Ang-(1–7), A779 and PD123319. Perindopril was given daily by intragastric intubation. The control and mock groups received the same volume of vehicle (NaCl 0.15 M) as the perindopril group by intragastric intubation and the same volume of vehicle as the Ang-(1–7) groups by subcutaneous administration via osmotic minipumps. The perindopril group received the same volume of vehicle as the Ang-(1–7) groups by subcutaneous administration via osmotic minipumps and the Ang-(1–7), Ang-(1–7)+A779, and Ang-(1–7)+PD123319 groups received the same volume of vehicle as the perindopril group by intragastric intubation. All rats were maintained on a normal diet for an additional 4 weeks and were then euthanatized after physiology studies. The left ventricle was excised for pathology and biochemistry. The study conforms to the Guide for the Care and Use of Laboratory Animals published by the US National Institutes of Health (NIH Publication, 8th Edition, 2011). The Institutional Animal Care and Use Committee at Qilu Hospital, Shandong University approved the protocol.

**Blood pressure measurement.** Heart rate, systolic blood pressure, diastolic blood pressure and mean arterial pressure were measured by use of a noninvasive tail-cuff device (Softron BP-98A; Softron, Tokyo, Japan) at the end of week 12 and 16. The rats were trained on three occasions before actual recordings were made and blood pressure measurements from three readings were averaged.

**Blood chemistry.** After fasting overnight, blood was withdrawn from the jugular vein in all rats. Fasting blood glucose level was analyzed by use of the Bayer 1650 blood chemistry analyzer (Bayer, Tarrytown, NY). Plasma angiotensin-converting enzyme 2 (ACE2) activity was determined by assay with internally quenched fluorescent substrate M-2660 (Abz-Ser-Pro-3-nitro-Tyr-OH, Bachem, Torrance, CA) as described^4^. The plasma activity of a disintegrin and metalloproteinase (ADAM) 17, also called tumor necrosis factor-α–converting enzyme, was assayed by use of the SensoLyte 520 ADAM17 Activity Assay Kit Fluorimetric (AnaSpec, San Jose, CA).

**Echocardiographic imaging.** Before and after 4-week treatment, rats in each group underwent transthoracic echocardiographic imaging. Transthoracic echocardiographic images were analyzed in a blinded manner as we described previously^2^ by use of a Vevo 770 high-resolution imaging system equipped with a 25-MHz transducer (RMV-710B, VisualSonics, Toronto, Canada). The following parameters were measured: intraventricular septal thickness, left ventricular (LV) posterior wall thickness, LV end-systolic diameter (LVESD) and volume (LVESV), and LV end-diastolic diameter (LVEDD) and volume (LVEDV). LV fractional shortening (FS) was calculated as: FS (%) = (LVEDD – LVESD)/LVEDD × 100% and LV ejection fraction (EF) as: LVEF (%) = (LVEDV – LVESV)/LVEDV × 100%. To assess LV diastolic function, the early (E-wave) and late (A-wave) diastolic mitral flow velocities were measured by pulsed-wave Doppler technique and the early (E') and late (A') diastolic mitral annular velocities were measured by tissue Doppler imaging. All measurements were averaged for three consecutive cardiac cycles by an experienced technician blinded to the rat grouping.

**Hemodynamic measurement.** At the end of weeks 12 and 16, rats in each group underwent cardiac catheterization. The Millar SPR-869 microtip catheter (Millar Instruments, Houston, Texas) connected to the PowerLab system (ADInstruments, Sydney, Australia) was introduced into the left ventricle via the right carotid artery to measure heart rate, maximal LV systolic pressure, LV end-diastolic pressure, maximal ascending rate of the LV pressure, and maximal descending rate of the LV pressure. Finally, the catheter was pulled back into the aorta to measure the mean arterial blood pressure.

**Preparation of paraffin-embedded tissue sections.** After physiology studies, rat hearts were perfuse-fixed with phosphate-buffered 4% paraformaldehyde, collected in ice-cold phosphate buffered saline (PBS), and bisected transversely at the midventricular level. Basal specimens were fixed in 4% paraformaldehyde overnight at 4 °C, washed with PBS, dehydrated through an ethanol series, embedded in paraffin, and cut into 4-µm thick sections.

**Histology.** Sections (4-µm thick) of paraffin-embedded hearts were stained with hematoxylin and eosin or Masson’s trichrome, and then visualized by light microscopy (Olympus, Tokyo) and photographed. The collagen components in a section were quantified by measuring the proportion of area positively stained with Masson’s trichrome to the total LV area by computer-assisted morphometry (ImagePro Plus v6.0, Media Cybernetics, Bethesda, MD). For each sample, all available fields (> 20 fields), visualized with a × 40 objective lens, were used for measurements.

As a measure of fibrosis, dark-green–stained collagen fibers were quantified in Masson trichrome-stained sections. The collagen volume fraction (CVF) and ratio of perivascular collagen area to luminal area (PVCA/LA) were analyzed by quantitative morphometry with ImagePro Plus 6.0. CVF was calculated as the area occupied by collagen divided by the total area in the visual field,^2^ with the perivascular collagen being excluded from the CVF measurement. To normalize the PVCA around vessels with different sizes, perivascular collagen content was represented as PVCA/LA.

**Transmission electron microscopy.** After hearts were excised, fresh LV tissue was quickly cut into 1-mm cubes, immersion-fixed in 2.5% glutaraldehyde in 0.1 M PBS (pH 7.4) overnight at 4 °C, and post-fixed in 1% buffered osmium tetroxide, and then dehydrated through a graded ethanol series and embedded in epoxy resin. Ultra-thin sections (90 nm) were double-stained with uranyl acetate and lead citrate, and examined by two pathologists blinded to the rat grouping using electron microscopy (model JEM-1200EX, Jeol Jem, Tokyo).

**Determination of cardiomyocyte cross-sectional area.** Sections of paraffin-embedded hearts were incubated with 5 μg·mL^-1^ Alexa Fluor® 488-conjugated wheat germ agglutinin (WGA; Invitrogen, Carlsbad, CA) in Hanks balanced salt solution (HBSS) in the dark for 10 min at room temperature. After a washing with PBS, LV cardiomyocyte membrane staining was examined by laser-scanning confocal microscopy (LSM710 Meta; Carl Zeiss, Jena, Germany). Myocyte cross-sectional area was quantified in 20 randomly chosen high-power fields in each section.

**Real-time RT-PCR.** Listed in Table S8 were primer sequences for real-time RT-PCR of rat collagen I-α1, collagen III-α1, brain natriuretic peptide, β-myosin heavy chain, fibronectin-1, transforming growth factor-beta 1 (TGF-β1), Bax, Bcl-2 and the housekeeping gene β-actin. Total RNA was extracted from freshly isolated myocardial samples by use of TriZol reagent (Invitrogen, Carlsbad, CA). Oligo (dT) primed cDNA synthesis involved use of Superscript III reverse transcriptase (Invitrogen, Carlsbad, CA). Transcripts were amplified from reverse-transcribed cDNA by use of SYBR Green (Invitrogen, Carlsbad, CA). Quantitative assessment of relative gene expression levels involved the 2^–△△CT^ method.

**Ang-(1–7), angiotensin-(1–9) [Ang-(1–9)] and angiotensin II (Ang-II) levels.** Plasma, tissue homogenates and cell lysates were stored at_80°C until extracted with C18 Sep-Pak cartridges (Waters Chromatography Division, Milford, MA) and assayed for Ang-(1–7), Ang-(1–9), and Ang-II levels by use of HPLC-based radioimmunoassay as described previously^3^.

**Myocardial ACE and ACE2 activities.** Myocardial ACE and ACE2 activities were determined by use of assays based on internally quenched fluorescent substrates (Abz-Phe-Arg-Lys (Dnp)-Pro-OH, M-2590 for ACE and Abz-Ser-Pro-3-nitro-Tyr-OH, M-2660 for ACE2, both from Bachem, Torrance, CA) with previously described methods^4,5^.

**Immunohistochemistry.** Tissue sections were prepared as described above, deparaffinized, and then incubated with a primary antibody against collagen I or III (both 1:100; both Abcam, Cambridge, UK), TGF-β1 (1:50; Bioworld, Louis Park, MN), Bax, Bcl-2 (both 1:50), IL-1β, IL-6 (both 1:100), or MCP-1 (1:200; all Abcam, Cambridge, UK) overnight at 4 °C. The addition of secondary antibody and color development followed the manufacturer’s instructions (Jingmei, Shenzhen, China). Nuclei were stained with hematoxylin. Data were analyzed by use of ImagePro Plus 6.0.

**Cell isolation, culture and treatment.** Primary cultures of the cardiac fibroblasts and myocytes in neonatal rats were performed as described^6^ with modifications. Briefly, neonatal Wistar rats (1 to 2 days old) were sacrificed by decapitation and the left ventricles were coarsely minced and predigested with 0.08% trypsin to remove erythrocytes, and then digested with 0.25% trypsin and 0.05% collagenase type 2 at 37 °C. The dissociated cells were placed in uncoated 100-mm culture discs and incubated at 37 °C in a 5% CO_2_ incubator for 1 hr, during which most of the cardiac fibroblasts rapidly adhered to the dishes. The attached cardiac fibroblasts were washed and further cultured in Dulbecco’s modified Eagle’s medium (DMEM; Gibco BRL, Gaithersburg, MD) supplemented with 100 units·mL^-1^ penicillin (Gibco BRL, Gaithersburg, MD), 100 units·mL^-1^ streptomycin (Gibco BRL, Gaithersburg, MD) and 10% fetal bovine serum (FBS; Gibco BRL, Gaithersburg, MD) at 37 °C in a 95% air/5% CO_2_ humidified atmosphere. The cells were split every 2 days with 0.25% trypsin PBS solution and cultured up to passage 3, and then plated at 2.5×10^4^ cells per well in 24-well tissue culture dishes in DMEM containing 100 units·mL^-1^ penicillin, 100 units·mL^-1^ streptomycin and 10% FBS in a humidified atmosphere of 95% air/5% CO_2_ at 37°C and grown to 85% confluence. Cells were serum starved for 24 hr before experiment.

The unattached cells were pre-plated for another hour on uncoated culture discs. Fibroblasts predominantly attached to dishes and most of the cardiomyocytes remained unattached. The population of cells enriched in cardiomyocytes was collected and counted. The cells were plated at 2.5×10^4^ cells/well in 24-well culture dishes and maintained in DMEM/F-12 containing 10% FBS, 100 units·mL^-1^ penicillin, 100 units·mL^-1^ streptomycin, and 0.1 mM bromodeoxyuridine under standard culture conditions. Using this protocol, 95% of the cells were deemed cardiomyocytes as judged by sarcomeric myosin content. On the second day after plating, medium was replaced. After 2 days, cells were incubated in serum-free medium containing transferrin (5 µg·mL^-1^), insulin (5 µg·mL^-1^), and 0.1 mM bromodeoxyuridine for 24 hr before treatment with indicated agents.

Cells were randomly divided into 10 groups for treatment: normal glucose, 5.6 mM glucose only; osmotic control, 5.6 mM glucose + 19.4 mM mannose; high glucose (HG), 25 mM glucose only; HG + perindopril group, 25 mM glucose + 10^-5^ M perindopril; HG + Ang-(1–7), 25 mM glucose + 10^-5^ M Ang-(1–7), HG + Ang-(1–7) + perindopril, 25 mM glucose + 10^-5^ M Ang-(1–7) + 10^-5^ M perindopril; HG + Ang-(1–7) + A779, 25 mM glucose + 10^-5^ M Ang-(1–7) + 10^-6^ M A779; HG + A779, 25 mM glucose + 10^-6^ M A779; HG + Ang-(1–7) + PD123319, 25 mM glucose + 10^-5^ M Ang-(1–7) + 10^-6^ M PD123319; and HG + PD123319, 25 mM glucose + 10^-6^ M PD123319. After exposure for 12, 24, 48 or 72 hr, cells and medium were collected for western blot and other analyses.

**Coculture of fibroblasts and myocytes.** Cardiac fibroblasts (2.5×10^4^ cells/well) were cocultured with myocytes (1.5×10^4^ cells/well) with or without Ang-(1–7) treatment for 72 hr at 37°C in the presence of 25 mM glucose in 24-well plates. In addition, cardiac fibroblasts were also incubated with the conditioned media of myocytes with or without Ang-(1–7) treatment for 72 hr in the presence of 25 mM glucose in 24-well plates. Thereafter, the expression of collagen I, collagen III and TGF-β1 protein in the media were determined by ELISA.

**^3^H-proline incorporation assay.** ^3^H-proline incorporation study was performed to investigate the effects of Ang-(1–7) on collagen synthesis in cardiac fibroblasts. Briefly, cardiac fibroblasts were treated with various agents for 72 hr: the first 24 hr of treatment maintained in DMEM containing 10% FBS; the second 24 hr under serum-free conditions; and the final 24 hr with 2.5% FBS and ^3^H-proline (1 μCi·mL^-1^). The experiments were terminated by washing cells twice with Dulbecco’s PBS and twice with ice-cold trichloroacetic acid (10%). The precipitate was dissolved in 500 mL of 0.3 N NaOH and 0.1% SDS after incubation at 50 °C for 2 hr. Aliquots from 4 wells for each treatment with 10 mL scintillation fluid were analyzed by use of a liquid scintillation counter.

**Collagen and TGF-β1 content by ELISA.** After incubation of cardiac fibroblasts with or without Ang-(1–7) or other agents, collagen I and III and TGF-β1 content was measured in the cell culture supernatant by use of three commercial ELISA kits (collagen I and collagen III rat ELISA kits from Uscnlife, Wuhan, China, and TGF-β1 rat ELISA kit from Abcam, Cambridge, UK).

**Immunocytochemistry.** Cells were allowed to grow in Lab-Tek chamber slides (Thermo Fisher Scientific, Fair Lawn, NJ) for immunocytochemistry, terminal deoxynucleotidyl transferase–mediated dUTP nick-end labeling (TUNEL) assay, and dihydroethidium (DHE) staining.

To evaluate the proliferative activity and the ability of cardiac fibroblasts to transform into myofibroblasts, we performed immunofluorescence staining for Ki67, a proliferative marker, and double immunofluorescence staining for S100A4 (a fibroblast marker) and α-smooth muscle actin (α-SMA; 1A4).

Fibroblasts were rinsed with PBS, fixed by PBS solution (pH 7.4) containing 4% paraformaldehyde for 30 min, permeabilized with 0.1% Triton X-100 for 5 min at room temperature, blocked with 5% bovine serum albumin (BSA) dissolved in PBS for 1 hr at 37 °C, and then incubated with a rabbit polyclonal antibody against Ki67 (Abcam, Cambridge, UK) at 1:50 overnight at 4 °C. After a washing, cells were treated with DyLight 549-conjugated goat anti-rabbit IgG (EarthOx, LLC, San Francisco, CA) at 1:500 for another 30 min. For determining myofibroblast transformation, a rabbit polyclonal antibody against S100A4 and a mouse monoclonal antibody against α-SMA were added (both 1:50; both Abcam, Cambridge, UK) in PBS containing 5% BSA for incubation overnight at 4 °C, then with the secondary antibodies DyLight 549-conjugated goat anti-rabbit IgG and DyLight™ 488-conjugated goat anti-mouse IgG (EarthOx, LLC, San Francisco, CA) (1:500 and 1:100, respectively) in PBS for 30 min at 37 °C.

Cardiomyocyte surface area was measured by immunocytochemistry with a rabbit polyclonal antibody against myosin heavy chain (MyHC; Santa Cruz Biotechnology, Santa Cruz, CA). Briefly, cardiomyocytes were rinsed with PBS, fixed with 4% paraformaldehyde for 30 min, permeabilized with 0.1% Triton X-100 for 5 min at room temperature, and blocked with 5% BSA for 1 hr at 37 °C, then incubated with a rabbit polyclonal antibody against MyHC (1:200) overnight at 4 °C. After a washing, cells were incubated with DyLight^TM^ 549-conjugated goat anti-rabbit IgG (1:500) for another 30 min at 37 °C.

Nuclei were stained with 4', 6-diamino-2-phenilindole (DAPI; Invitrogen, Carlsbad, CA) for 10 min at room temperature. The laser-scanning confocal image system as described above was used for analysis.

**Preparation of frozen myocardial sections.** After anaesthesia with pentobarbital, rats were euthanatized. Rat hearts were perfusion-fixed with 4% paraformaldehyde, 5% sucrose and 20 mM EDTA (pH 7.4) for 10 min, then excised and embedded in optimal-cutting-temperature compound, quick-frozen on dry ice, and stored at -80 °C. Serial 10-μm–thick myocardial sections were obtained by use of a sliding microtome (Leica CM1900, Nussloch, Germany).

**Detection and quantitation of apoptosis.** Apoptotic cells in tissue sections were quantified by TUNEL staining with a CardioTACS Kit (Trevigen, Gaithersburg, MD) according to the supplier's protocol. Apoptosis of cultured cardiomyocytes was evaluated by double immunofluorescence for MyHC and TUNEL (Roche, Mannheim, Germany).

**DHE fluorescence.** The oxidative fluorescent dye DHE was used to measure superoxide (O_2_^–^) levels in rat heart tissues and cultured cardiac fibroblasts and myocytes as described previously^7^. Briefly, 10-μm fresh frozen myocardial sections were washed with HBSS with magnesium and calcium, and then incubated at 37 °C for 30 min with DHE (10 μM) in HBSS. For cultured and treated cardiac fibroblasts and myocytes, cells were washed with clear media (DMEM), and incubated at 37 °C for 30 min with DHE (10 μM) in clear media. One tissue slide or one cell plate without DHE staining served as a blank control. The tissue slides or cell plates were wrapped with foil to minimize exposure to light. Fluorescent images were observed and analyzed by confocal microscopy as described above.

**Lucigenin-enhanced chemiluminescence.** The activities of NADPH oxidase in rat hearts and cultured cardiac fibroblasts and myocytes were quantified by lucigenin-enhanced chemiluminescence as reported previously^7^. Briefly, cell and heart homogenates (200-μg total proteins) were collected in 100 μl of PBS mixture with phosphatase and protease inhibitors and then centrifuged at 1,000 rpm for 10 min. Supernatants were collected and added to NADPH (1 mM) and lucigenin (50 μM) for NADPH oxidase activity assay by FB-12 luminometry with or without diphenylene iodonium (10 μM), a selective inhibitor of flavin-containing enzymes, including NADPH oxidase. Data were calculated as the change in the rate of luminescence per min per milligram of tissues or cells.

**Western blot analysis.** Proteins from tissue homogenates and cell lysates were extracted for western blot analysis. After quantification by use of the BCA Protein Array Kit (Pierce, Rockford, IL), the soluble protein fraction was resuspended in Laemmli loading buffer (2% SDS, 20% glycerol, 0.04 mg·mL^-1^ bromophenol blue, 0.12 M Tris·HCl, pH 6.8, and 0.28 M β-mercaptoethanol). Equal amounts of protein (30 µg) were separated by 10%~15% SDS-PAGE by the Bio-Rad Mini-Protein II system (90 and 110 V during the stacking and separation gels, respectively), then transferred to polyvinylidene difluoride membranes (Millipore, Billerica, MA) by the Bio-Rad Trans-Blot system (1.5 hr at 200 mA in 25 mM Tris, 192 mM glyceine, and 20% MeOH). Membranes were blocked with 5% skimmed milk powder dissolved in Tris–Buffered Saline Tween-20 (TBST) (10 mM Tris, 0.1 M NaCl, 0.1% Tween 20, pH 7.4) for 2 hr, then incubated overnight at 4 °C with gentle agitation with primary antibodies against TGF-β1 (44 kDa; Bioworld, Louis Park, MN), MasR (50 kDa; Alomone Labs, Jerusalem, Israel), Bax (20 kDa), Bcl-2 (26 kDa), angiotensin II type 1 receptor (AT_1_R) (38 kDa), AT_2_R (44 kDa), β-actin (42 kDa), and total and phosphorylated extracellular signal-regulated kinase 1/2 (ERK1/2) (44/42 kDa) and p38 mitogen-activated protein kinase (p38-MAPK) (38 kDa) (all Abcam, Cambridge, UK). Following incubation with primary antibodies, membranes were washed 3 times for 15 min each with TBST buffer, incubated with appropriate horseradish peroxidase-labeled secondary antibodies (Abcam, Cambridge, UK) in 5% milk in TBST buffer for 2 hr at room temperature, then rinsed thoroughly with TBST buffer. The bands were developed by use of enhanced chemiluminescent reagent (Amersham Pharmacia Biotech, Piscataway, NJ), detected by AlphaImager HP, and quantified by densitometry with ImageJ (US National Institutes of Health, Bethesda, MD). Signals from phosphor-proteins were normalized to their total proteins. Other protein levels were normalized to that of β-actin as an internal control.

**Statistical analysis.** SPSS v11.5 (SPSS Inc., Chicago, IL) was used for analysis. Continuous data are expressed as mean ± SEM and were compared by one-way ANOVA, followed by Tukey–Kramer post-hoc test and independent samples *t* test. *P* < 0.05 was considered statistically significant.

**Supplementary references**

1. Dong B, *et al*. Angiotensin-converting enzyme-2 overexpression improves left ventricular remodeling and function in a rat model of diabetic cardiomyopathy. *J Am Coll Cardiol* **59,** 739 (2012).

2. Ti Y, *et al*. TRB3 gene silencing alleviates diabetic cardiomyopathy in a type 2 diabetic rat model. *Diabetes* **60,** 2963 (2011).

3. Campbell DJ, Krum H, Esler MD. Losartan increases bradykinin levels in hypertensive humans. *Circulation* **111,** 315 (2005).

4. Yan ZH, *et al*. Development of intramolecularly quenched fluorescent peptides as substrates of angiotensin-converting enzyme 2. *Anal Biochem* **312,** 141 (2003).

5. Alves MF, *et al*. A continuous fluorescent assay for the determination of plasma and tissue angiotensin I-converting enzyme activity. *Braz J Med Biol Res* **38,** 861 (2005).

6. van Kesteren CA, *et al*. Cultured neonatal rat cardiac myocytes and fibroblasts do not synthesize renin or angiotensinogen: evidence for stretch-induced cardiomyocyte hypertrophy independent of angiotensin II. *Cardiovasc Res* **43,** 148 (1999).

7. Zhong J, *et al*. Angiotensin-converting enzyme 2 suppresses pathological hypertrophy, myocardial fibrosis, and cardiac dysfunction. *Circulation* **122,** 717 (2010).

**Supplementary figures**

Supplementary Figure 1. Representative echocardiographic images in 9 groups of rats. (A) M-mode echocardiographic images of the left ventricles. (B) Pulsed-wave Doppler recordings of the mitral inflow. (C) Tissue Doppler recordings of the mitral annulus. A200: low-dose Ang-(1–7) (200 ng·kg^-1^·min^-1^), A400: moderate-dose Ang-(1–7) (400 ng·kg^-1^·min^-1^), A800: high-dose Ang-(1–7) (800 ng·kg^-1^·min^-1^), P: perindopril.


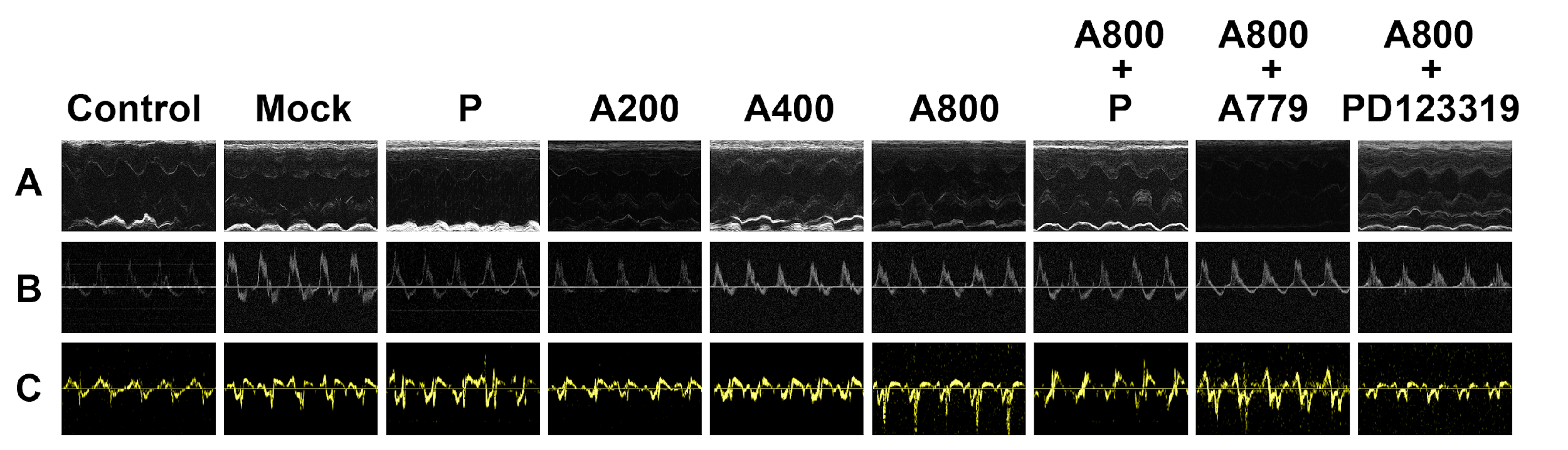


Supplementary Figure 2. Ang-(1–7) suppresses oxidative stress and inflammation in the myocardium of diabetic rats. (A) Representative dihydroethidium (DHE) fluorescence images (scale bar: 50 µm) and immunohistochemical staining for myocardial IL-1β, IL-6 and MCP-1 (scale bar: 50 µm) in 9 groups of rats. (B) Quantification of DHE fluorescence values and NADPH oxidase activity. (C) Quantification of positive IL-1β, IL-6 and MCP-1 staining in 9 groups of rats. **P* < 0.05 and ***P* < 0.01 vs. control group; ^#^*P* < 0.05 and ^##^*P* < 0.01 vs. mock; ^†^*P* < 0.05 and ^††^*P* < 0.01 vs. perindopril (P); ^&^*P* < 0.05 and ^&&^*P* < 0.01 vs. high-dose Ang-(1–7) (800 ng·kg^-1^·min^-1^) (A800). A200: low-dose Ang-(1–7) (200 ng·kg^-1^·min^-1^), A400: moderate-dose Ang-(1–7) (400 ng·kg^-1^·min^-1^).


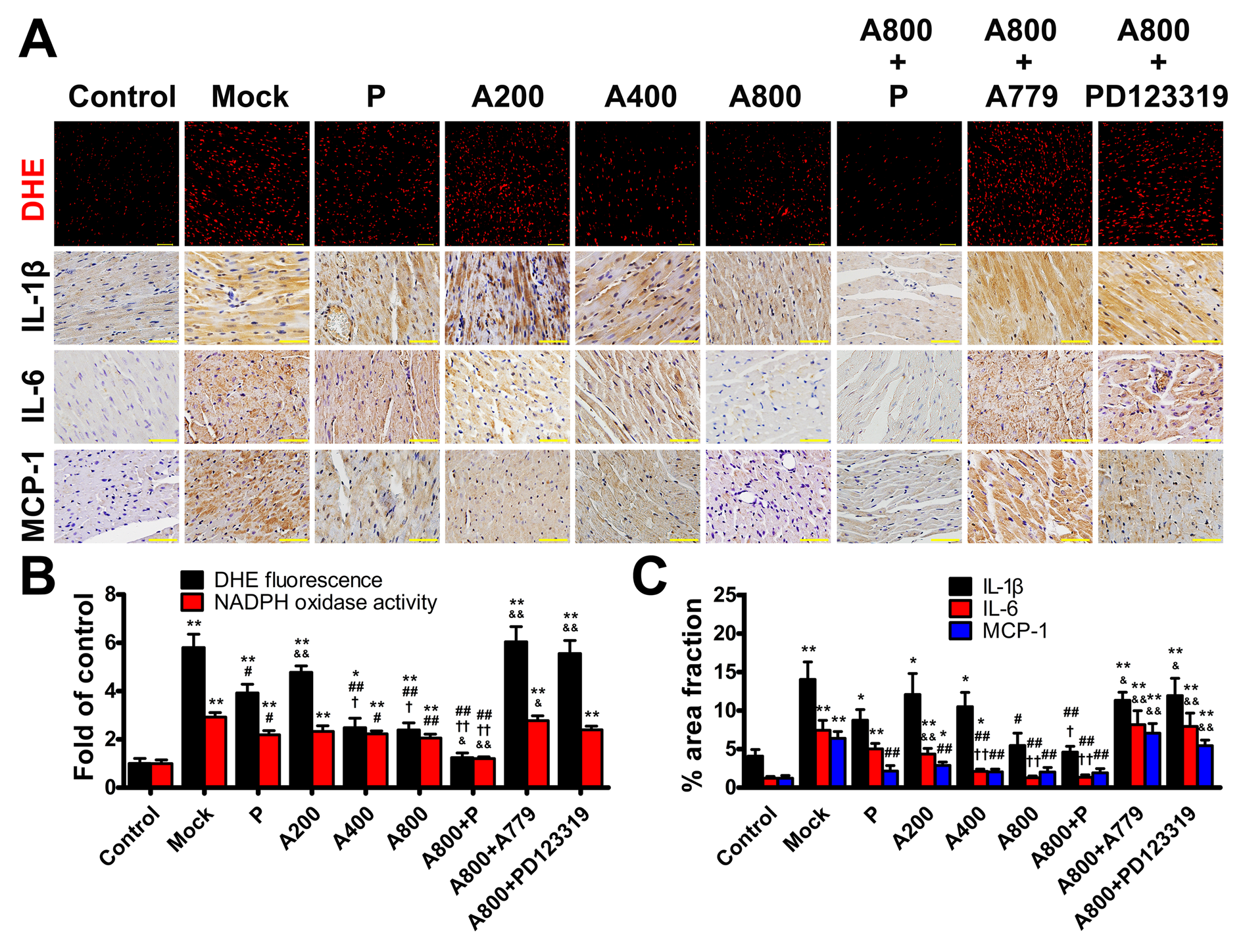


Supplementary Figure 3. Ang-(1–7) treatment attenuates collagen expression in cardiac fibroblasts. ELISA of time-response of collagen I (Coll I) (A) and collagen III (Coll III) (B) protein expression in cultured media of cardiac fibroblasts after high-glucose (HG) stimulation and single-dose Ang-(1–7) treatment. **P* < 0.05 vs. normal glucose (NG); ^#^*P* < 0.05 vs. osmotic control (OC); ^†^*P* < 0.05 vs. HG. ELISA of dose-response of Coll I (C) and Coll III (D) protein expression in cultured media of cardiac fibroblasts after HG stimulation and Ang-(1–7) treatment at 3 doses. **P* < 0.05, ***P* < 0.01. ELISA of Coll I (E) and Coll III (F) protein expression in cultured media of cardiac fibroblasts after treatments. (G) Quantification of the ratio of Coll I to Coll III protein expression after treatments. (H) Collagen synthesis determined by ^3^H-proline incorporation in cardiac fibroblasts after treatments. ***P* < 0.01 vs. NG; ^#^*P* < 0.05 and ^##^*P* < 0.01 vs. HG; ^†^*P* < 0.05 and ^††^*P* < 0.01 vs. HG+perindopril (P); ^&&^*P* < 0.01 vs. HG+Ang-(1–7) (A).


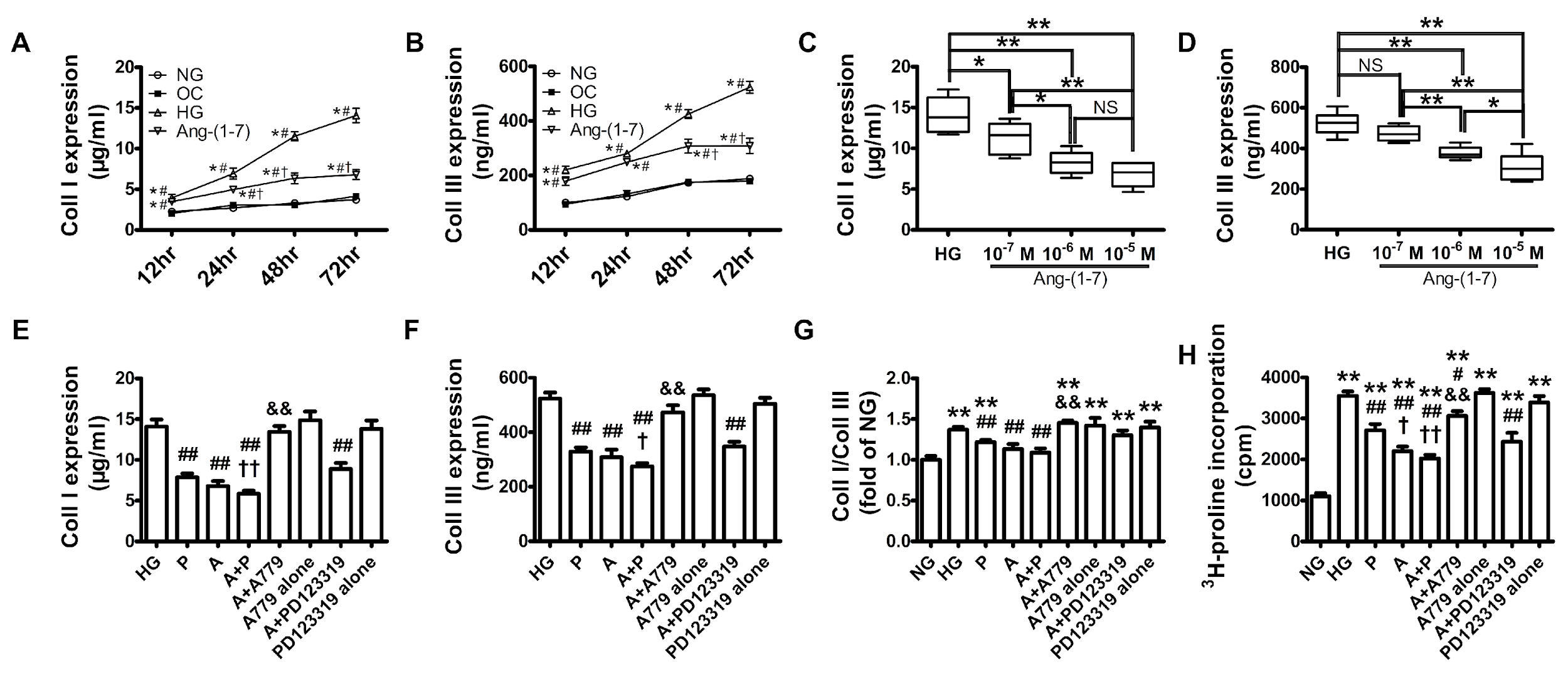


Supplementary Figure 4. Ang-(1–7) suppresses fibroblast–myocyte communication. ELISA of collagen I (Coll I) (A), collagen III (Coll III) (B) and TGF-β1 (C) levels in fibroblasts (F), cocultured fibroblasts and Ang-(1–7)-non-treated myocytes (F+M), cocultured fibroblasts and Ang-(1–7)-treated myocytes (F+M-A), cocultured fibroblasts and Ang-(1–7) plus A779-treated myocytes (F+M-AA) and cocultured fibroblasts and Ang-(1–7) plus PD123319-treated myocytes (F+M-APD). **P* < 0.05 and ***P* < 0.01 vs. F; ^#^*P* < 0.05 and ^##^*P* < 0.01 vs. F+M; ^†^*P* < 0.05 and ^††^*P* < 0.01 vs. F+M-A. ELISA of Coll I (D), Coll III (E) and TGF-β1 (F) levels in fibroblasts (F) and fibroblasts incubated with the conditioned media of Ang-(1–7)-non-treated myocytes (F+Me), Ang-(1–7)-treated myocytes (F+Me-A), Ang-(1–7) plus A779-treated myocytes (F+Me-AA) and Ang-(1–7) plus PD123319-treated myocytes (F+Me-APD). **P* < 0.05 and ***P* < 0.01 vs. F; ^#^*P* < 0.05 and ^##^*P* < 0.01 vs. F+Me; ^†^*P* < 0.05 and ^††^*P* < 0.01 vs. F+Me-A.


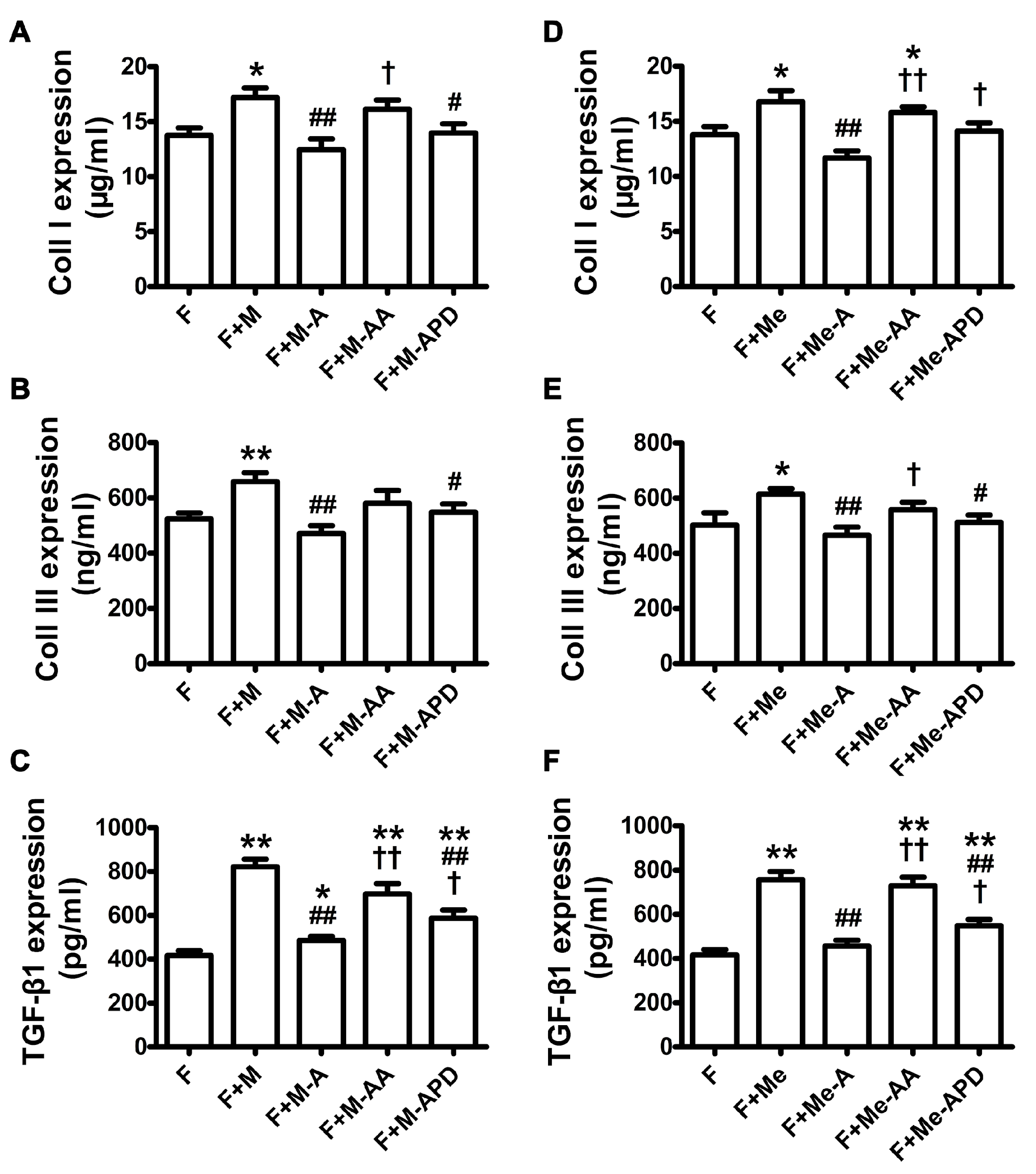


Supplementary Figure 5. Ang-(1–7) treatment prevents cardiomyocyte hypertrophy and apoptosis induced by HG *in vitro*. (A) Representative TUNEL staining (green) with counterstaining for myosin heavy chain (MyHC) (red) and nuclei (DAPI; blue) in cardiac myocytes (scale bar: 50 µm). Quantification of (B) cardiomyocyte surface area after different treatments; (C) rate of myocyte apoptosis indicated by TUNEL-positive cells as a percentage of total MyHC-positive cells after different treatments, (D) dihydroethidium (DHE) fluorescence and (E) NADPH oxidase activity in cardiac myocytes with different treatments. **P* < 0.05 and ***P* < 0.01 vs. NG; ^#^*P* < 0.05 and ^##^*P* < 0.01 vs. HG; ^†^*P* < 0.05 vs. HG+perindopril (P); ^&^*P* < 0.05 and ^&&^*P* < 0.01 vs. HG+Ang-(1–7) (A).


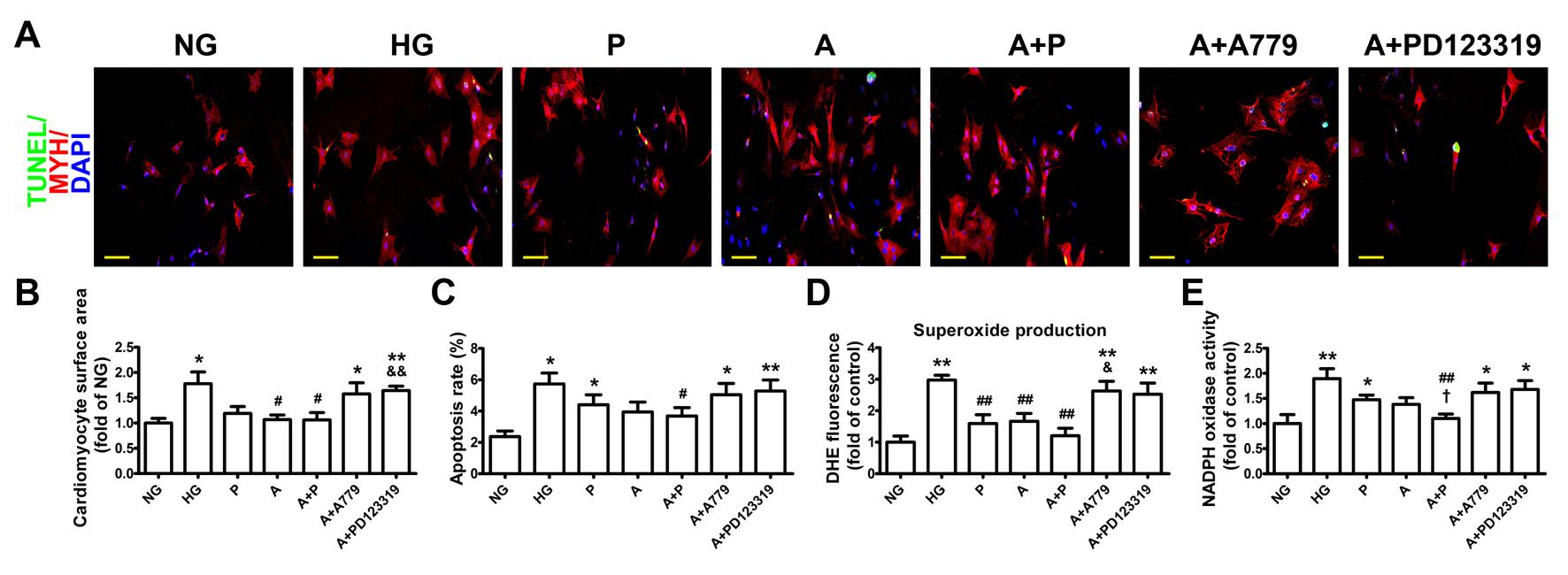


Supplementary Figure 6. Ang-(1–7) treatment has no effect on Ang-II levels *in vitro*. Ang-II levels in cardiac fibroblasts (A) and myocytes (B) after treatments. **P* < 0.05 and ***P* < 0.01 vs. NG; ^#^*P* < 0.05 vs. HG; ^†^*P* < 0.05 vs. HG+P; ^&^*P* < 0.05 and ^&&^*P* < 0.01 vs. HG+Ang-(1–7) (A).


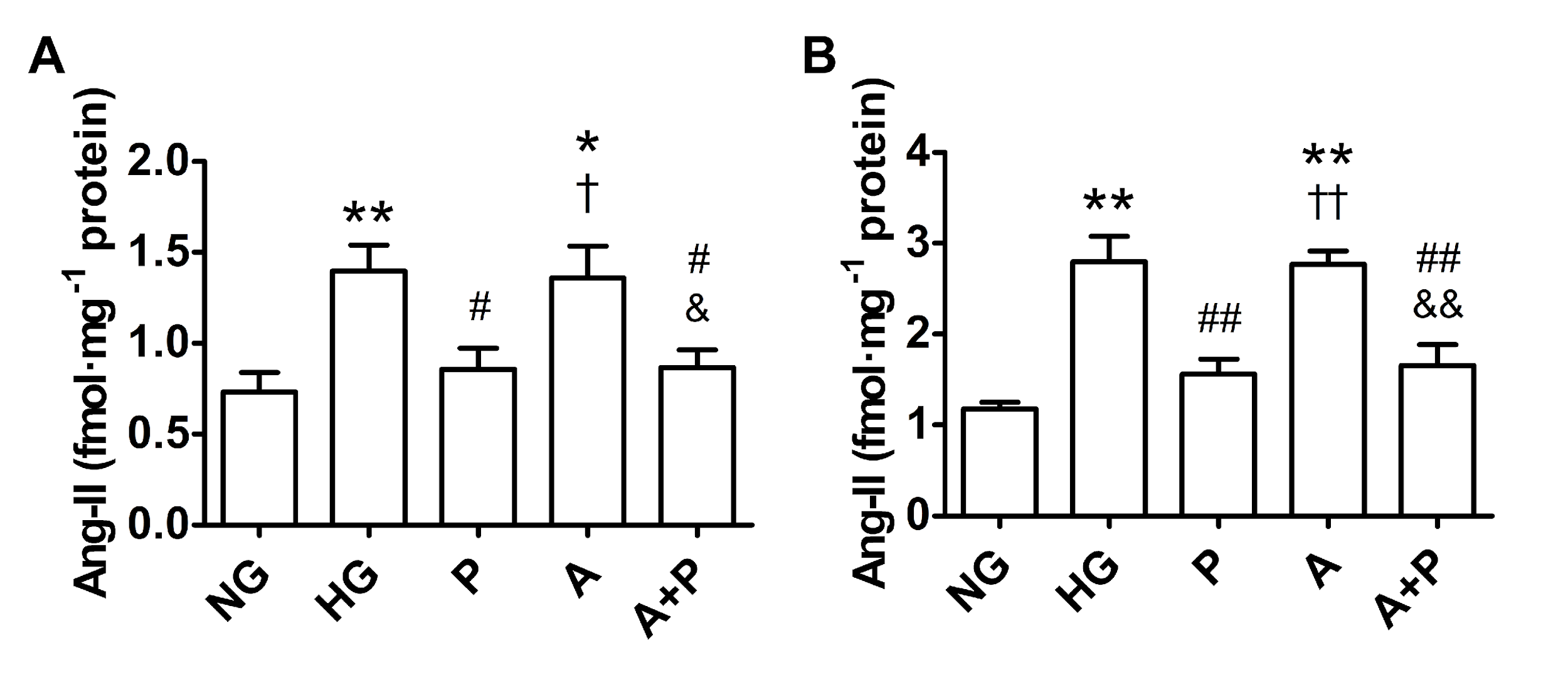


Supplementary Figure 7. Ang-(1–7) increases angiotensin-converting enzyme 2 (ACE2) activity in the myocardium of diabetic rats. Quantification of activities of ACE (A) and ACE2 (B) in the rat myocardium. **P* < 0.05 and ***P* < 0.01 vs. control; ^#^*P* < 0.05 and ^##^*P* < 0.01 vs. mock; ^†^*P* < 0.05 vs. P; ^&^*P* < 0.05 vs. high-dose Ang-(1–7) (800 ng·kg^-1^·min^-1^) (A800).


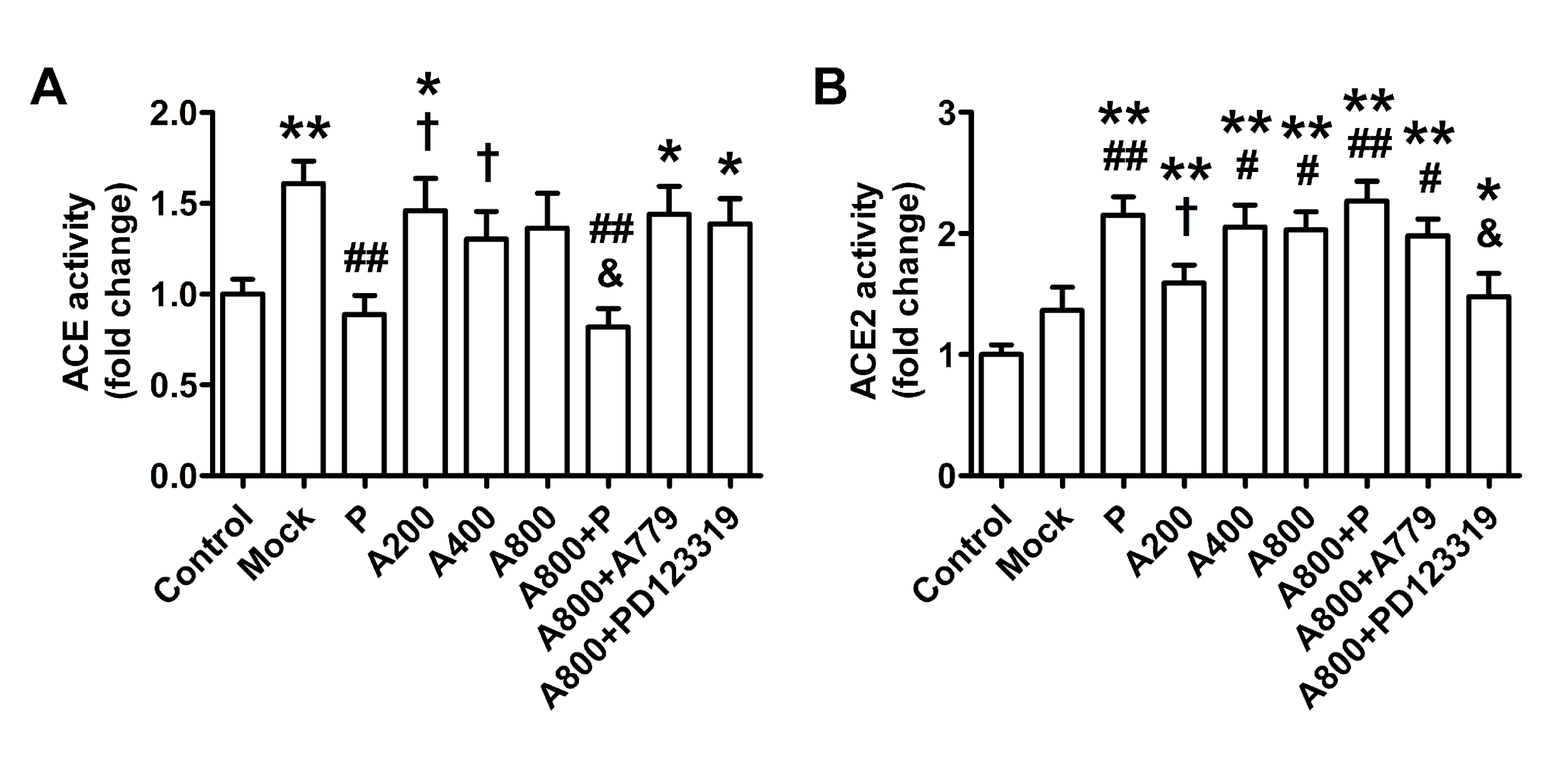


Supplementary Figure 8. Ang-(1–7) increases plasma ACE2 activity in diabetic rats. Quantification of activity of a disintegrin and metalloproteinase 17 (ADAM17) (A) and ACE2 (B) in plasma with treatments. **P* < 0.05 and ***P* < 0.01 vs. control; ^#^*P* < 0.05 and ^##^*P* < 0.01 vs. mock; ^†^*P* < 0.05 and ^††^*P* < 0.01 vs. P; ^&^*P* < 0.05 and ^&&^*P* < 0.01 vs. high-dose Ang-(1–7) (800 ng·kg^-1^·min^-1^) (A800).


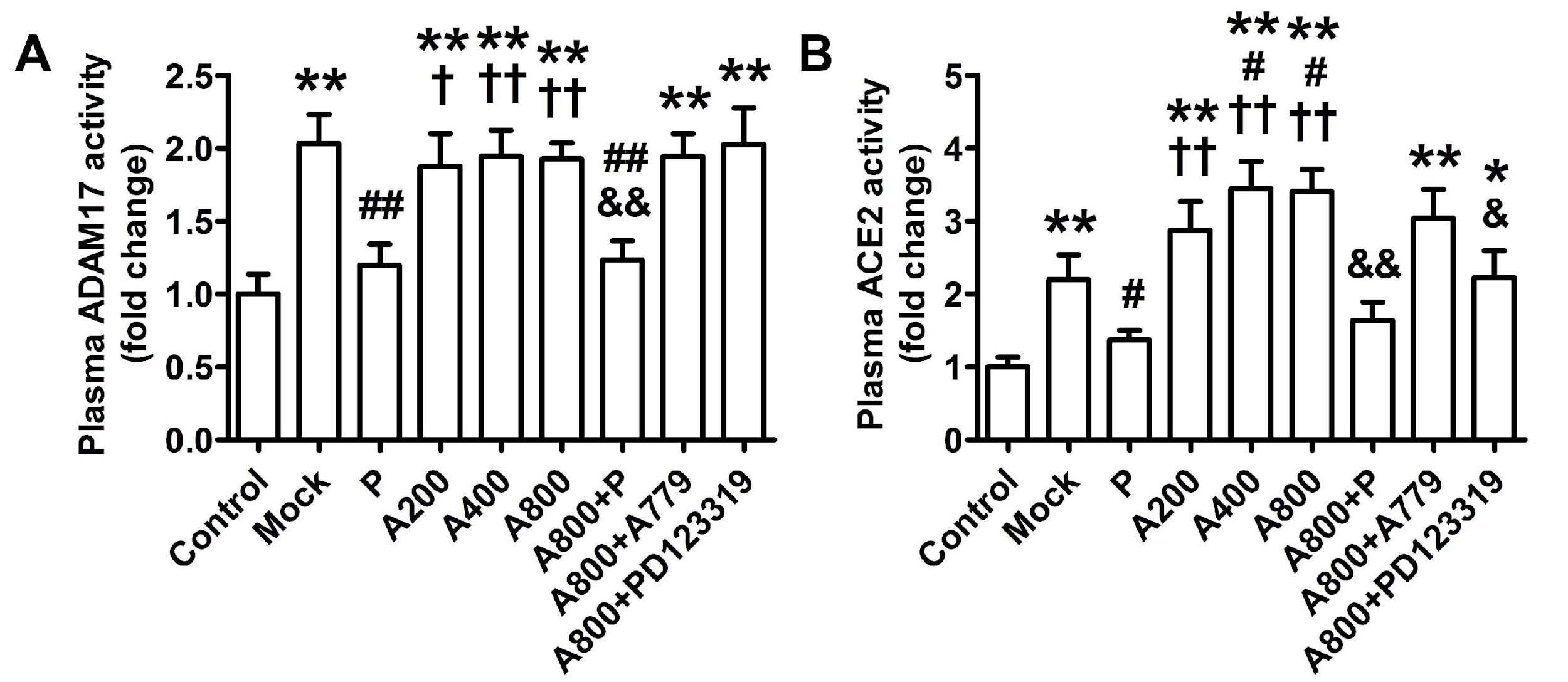


Supplementary Figure 9. Proposed mechanism underlying the therapeutic effects of Ang-(1–7) in a rat model of diabetic cardiomyopathy. AT_1_R: Ang-II type 1 receptor, AT_2_R: Ang-II type 2 receptor, HG: high glucose, MasR: Mas receptor.


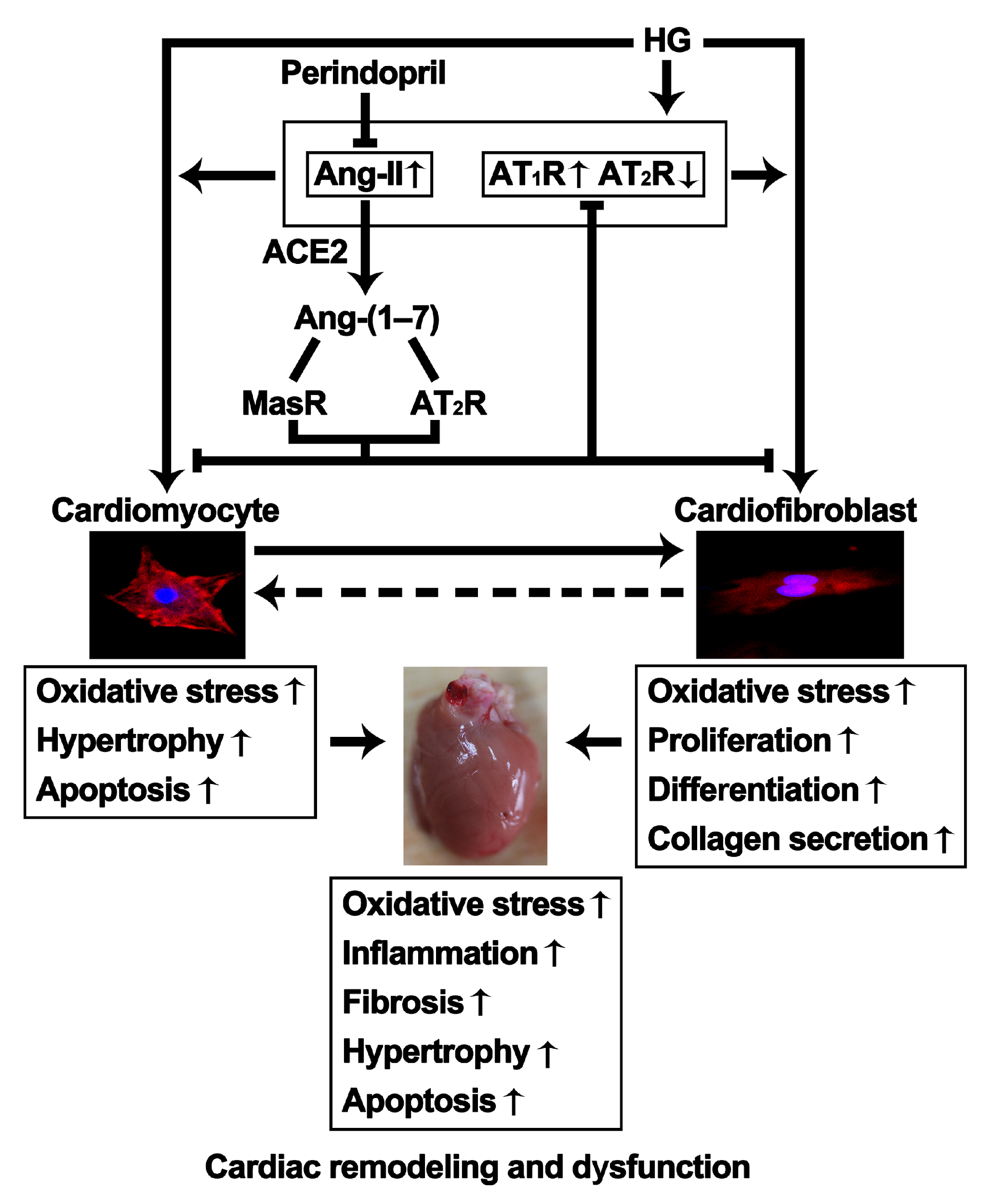


**Supplementary tables**

Supplementary Table 1. Primer sequences for real-time RT-PCR.

| Gene | Forward Primer (5′-3′) | Reverse Primer (5′-3′) | Product length (bp) |
| --- | --- | --- | --- |
| β-actin | CCTGTACGCCAACACAGTGC | ATACTCCTGCTTGCTGATCC | 211 |
| Coll Iα1 | CTACAGCACGCTTGTGGATG | CAGATTGGGATGGAGGGAGT | 195 |
| Coll IIIα1 | CCTCCCAGAACATTACATACCAC | GACTGTCTTGCTCCATTCACCA | 194 |
| BNP | GGGCTGTGACGGGCTGA | TGTGGCAAGTTTGTGCTGGA | 200 |
| β-MHC | AGGCCGAGTTCCAGAAGATG | TGCTTCACCCGCTGTAGATT | 142 |
| Fibronectin-1 | TCGCTTTGACTTCACCACCAG | CCTCGCTCAGTTCGTACTCCA | 207 |
| TGF-β1 | GCTGAACCAAGGAGACGGAATA | ACCTCGACGTTTGGGACTGA | 115 |
| Bax | TGCTACAGGGTTTCATCCAG | ATCCACATCAGCAATCATCC | 175 |
| Bcl-2 | TGGGATGCCTTTGTGGAAC | CATATTTGTTTGGGGCAGGTC | 167 |

BNP: brain natriuretic peptide, Coll Iα1: collagen I-α1, Coll IIIα1: collagen III-α1, β-MHC: β-myosin heavy chain, TGF-β1: transforming growth factor-beta 1.

Supplementary Table 2. Characteristics of 9 groups of rats before 4-week treatment.

|  | Control | Mock | P | A200 | A400 | A800 | A800+P | A800+A779 | A800+PD123319 |
| --- | --- | --- | --- | --- | --- | --- | --- | --- | --- |
| HR (bpm) | 371±15 | 364±12 | 363±19 | 356±21 | 366±11 | 355±24 | 363±17 | 359±16 | 353±14 |
| SBP (mmHg) | 122±6 | 111±5 | 114±3 | 107±5 | 109±4 | 113±4 | 110±7 | 106±5 | 108±6 |
| DBP (mmHg) | 95±5 | 83±3 | 82±7 | 81±3* | 85±5 | 82±4 | 80±7 | 84±3 | 86±4 |
| MAP (mmHg) | 110±5 | 97±4 | 98±4 | 94±3* | 96±2* | 97±5 | 95±6 | 96±4* | 99±5 |
| FBG (mM) | 3.52±0.37 | 24.23±1.37* | 23.58±1.20* | 24.19±1.03* | 23.31±0.84* | 24.38±1.33* | 25.37±1.64* | 24.72±1.06* | 23.53±0.92* |

A200: Ang-(1–7) at 200 ng·kg^-1^·min^-1^, A400: Ang-(1–7) at 400 ng·kg^-1^·min^-1^, A800: Ang-(1–7) at 800 ng·kg^-1^·min^-1^, DBP: diastolic blood pressure, FBG: fasting blood glucose, HR: heart rate, MAP: mean arterial pressure, P: perindopril, SBP: systolic blood pressure. **P* < 0.05 vs. control group.

Supplementary Table 3. Characteristics of 9 groups of rats after 4-week treatment.

|  | Control | Mock | P | A200 | A400 | A800 | A800+P | A800+A779 | A800+PD123319 |
| --- | --- | --- | --- | --- | --- | --- | --- | --- | --- |
| HR (bpm) | 365±8 | 360±9 | 359±7 | 353±7 | 357±10 | 360±18 | 360±15 | 364±13 | 363±10 |
| SBP (mmHg) | 124±2 | 106±3* | 92±4*^#^ | 106±3*^†^ | 101±4* | 102±4* | 95±5*^#^ | 103±4* | 110±3* |
| DBP (mmHg) | 96±3 | 81±3* | 68±5*^#^ | 82±4*^†^ | 79±3*^†^ | 78±4* | 66±4*^#&^ | 76±4* | 80±2* |
| MAP (mmHg) | 107±2 | 93±2* | 79±2*^#^ | 94±3*^†^ | 90±2*^†^ | 90±3*^†^ | 79±2*^#&^ | 88±2* | 93±2* |
| FBG (mM) | 3.76±0.23 | 22.99±0.97* | 22.57±1.13* | 23.10±0.75* | 22.35±1.16* | 23.01±1.24* | 23.10±1.20* | 23.05±1.00* | 23.18±1.05* |

Abbreviations as in Supplementary Table 2. **P* < 0.05 vs. control group; ^#^*P* < 0.05 vs. mock group; ^†^*P* < 0.05 vs. perindopril (P) group; ^&^*P* < 0.05 vs. high-dose Ang-(1–7) (800 ng·kg^-1^·min^-1^) (A800) group.

Supplementary Table 4. Echocardiographic assessment of cardiac structure and function before 4-week treatment.

|  | Control | Mock | P | A200 | A400 | A800 | A800+P | A800+A779 | A800+PD123319 |
| --- | --- | --- | --- | --- | --- | --- | --- | --- | --- |
| IVSth (mm) | 1.33±0.05 | 1.87±0.09* | 1.90±0.09* | 1.93±0.06* | 1.94±0.07* | 1.83±0.09* | 1.88±0.12* | 1.86±0.07* | 1.90±0.10* |
| LVPWth (mm) | 1.32±0.05 | 1.96±0.11* | 2.01±0.09* | 1.98±0.08* | 2.12±0.10* | 1.93±0.09* | 1.94±0.10* | 2.15±0.12* | 1.90±0.12* |
| LVESD (mm) | 2.90±0.04 | 4.25±0.09* | 4.28±0.12* | 4.28±0.12* | 4.41±0.11* | 4.28±0.11* | 4.20±0.15* | 4.18±0.13* | 4.33±0.12* |
| LVEDD (mm) | 4.89±0.05 | 6.43±0.16* | 6.42±0.15* | 6.40±0.17* | 6.50±0.16* | 6.36±0.20* | 6.29±0.20* | 6.19±0.18* | 6.38±0.14* |
| LVEF (%) | 71.29±0.86 | 61.44±1.01* | 60.86±1.05* | 60.34±1.65* | 59.15±1.18* | 59.55±1.22* | 60.69±1.19* | 59.63±1.41* | 59.05±2.01* |
| FS (%) | 40.76±0.74 | 33.79±0.76* | 33.37±0.73* | 33.08±1.21* | 32.19±0.85* | 32.43±0.90* | 33.20±0.84* | 32.44±1.00* | 32.21±1.41* |
| E/A ratio | 2.03±0.06 | 1.27±0.07* | 1.22±0.08* | 1.16±0.08* | 1.24±0.10* | 1.14±0.06* | 1.12±0.11* | 1.21±0.10* | 1.23±0.07* |
| E′/A′ ratio | 1.65±0.05 | 0.90±0.06* | 0.90±0.05* | 0.89±0.05* | 0.93±0.05* | 0.90±0.06* | 0.90±0.06* | 0.89±0.05* | 0.95±0.06* |

E/A ratio: ratio of early to late left ventricular filling velocity, E'/A' ratio: ratio of early to late diastolic peak annular velocity, FS: fraction shortening, IVSth: intraventricular septal thickness, LVEDD: left ventricular end diastolic diameter, LVEF: left ventricular ejection fraction, LVESD = left ventricular end systolic diameter, LVPWth: left ventricular posterior wall thickness, other abbreviations as in Supplementary Table 2. **P* < 0.05 vs. control group.

Supplementary Table 5. Hemodynamic assessment of cardiac function before 4-week treatment.

|  | Control | Mock | P | A200 | A400 | A800 | A800+P | A800+A779 | A800+PD123319 |
| --- | --- | --- | --- | --- | --- | --- | --- | --- | --- |
| LVSP (mmHg) | 124.3±2.5 | 108.8±3.5* | 109.8±3.7* | 105.4±4.0* | 112.6±3.9* | 109.4±3.5* | 109.1±4.8* | 107.6±2.4* | 112.5±3.0* |
| LVEDP (mmHg) | 6.38±0.60 | 10.50±0.42* | 10.50±0.73* | 10.25±0.59* | 10.75±0.70* | 10.88±0.83* | 10.75±0.75* | 10.88±0.81* | 11.13±0.52* |
| +dp/dt (mmHg/sec) | 6870±267 | 5824±205* | 5956±148* | 5613±166* | 5887±167* | 5893±307* | 5697±245* | 5829±270* | 5716±264* |
| -dp/dt (mmHg/sec) | 5124±88 | 4475±97* | 4464±73* | 4445±113* | 4452±129* | 4530±128* | 4420±114* | 4461±92* | 4459±82* |

+dp/dt: maximal rate of pressure rise, -dp/dt: maximal rate of pressure fall, LVEDP: left ventricular end diastolic pressure, LVSP: left ventricular systolic pressure, other abbreviations as in Supplementary Table 2. **P* < 0.05 vs. control group.

Supplementary Table 6. Plasma levels of Ang-(1-7), Ang-(1-9), and Ang-II before 4-week treatment.

|  | Control | Mock | P | A200 | A400 | A800 | A800+P | A800+A779 | A800+PD123319 |
| --- | --- | --- | --- | --- | --- | --- | --- | --- | --- |
| Ang-(1–7) (fmol/mL) | 29.19±3.60 | 21.99±2.87 | 23.51±3.94 | 22.46±3.72 | 22.33±3.32 | 25.56±4.18 | 25.92±3.08 | 25.19±3.29 | 19.94±2.72 |
| Ang-(1–9) (fmol/mL) | 7.17±0.94 | 9.84±0.78 | 9.19±0.96 | 9.84±1.19 | 8.92±1.05 | 9.50±0.92 | 10.01±1.51 | 9.18±0.83 | 9.36±0.77 |
| Ang-II (fmol/mL) | 60.41±4.07 | 94.06±7.60* | 98.03±9.40* | 92.24±9.77* | 98.85±8.01* | 90.21±6.67* | 99.77±9.03* | 99.75±9.51* | 89.60±6.34* |

Abbreviations as in Supplementary Table 2. **P* < 0.05 vs. control group.

Supplementary Table 7. Plasma levels of Ang-(1-7), Ang-(1-9), and Ang-II after 4-week treatment.

|  | Control | Mock | P | A200 | A400 | A800 | A800+P | A800+A779 | A800+PD123319 |
| --- | --- | --- | --- | --- | --- | --- | --- | --- | --- |
| Ang-(1–7) (fmol/mL) | 27.67±3.09 | 18.28±2.26* | 25.96±3.10 | 100.4±17.04*^#†&^ | 143.4±27.10*^#†^ | 177.5±21.67*^#†^ | 234.1±25.94*^#†^ | 163.6±14.77*^#^ | 180.1±18.79*^#^ |
| Ang-(1–9) (fmol/mL) | 7.49±0.76 | 10.26±1.04 | 27.83±3.58*^#^ | 11.81±1.00*^†^ | 14.00±1.69*^†^ | 15.24±1.53*^#†^ | 31.38±3.73*^#&^ | 15.68±1.46*^#^ | 13.39±2.12* |
| Ang-II (fmol/mL) | 66.63±3.17 | 105.5±10.78* | 61.14±±4.83^#^ | 92.19±6.91*^†^ | 86.80±5.44*^†^ | 82.53±6.65^†^ | 64.88±5.93^#^ | 92.87±10.84* | 100.7±11.99* |

Abbreviations as in Supplementary Table 2. **P* < 0.05 vs. control group; ^#^*P* < 0.05 vs. mock group; ^†^*P* < 0.05 vs. perindopril (P) group; ^&^*P* < 0.05 vs. high-dose Ang-(1–7) (800 ng·kg^-1^·min^-1^) (A800) group.

Supplementary Table 8. Myocardial levels of Ang-(1-7), Ang-(1-9), and Ang-II after 4-week treatment.

|  | Control | Mock | P | A200 | A400 | A800 | A800+P | A800+A779 | A800+PD123319 |
| --- | --- | --- | --- | --- | --- | --- | --- | --- | --- |
| Ang-(1–7) (fmol/mg) | 4.91±0.39 | 2.91±0.37* | 3.39±0.35* | 7.31±0.63*^#†&^ | 11.21±1.15*^#†^ | 12.98±1.88*^#†^ | 18.35±1.67*^#†^ | 12.84±1.42*^#^ | 13.79±1.02*^#^ |
| Ang-(1–9) (fmol/mg) | 1.91±0.21 | 2.40±0.21 | 4.30±0.27*^#^ | 2.52±0.30^†^ | 2.90±0.25*^†^ | 3.23±0.26*^#†^ | 4.59±0.29*^#&^ | 3.10±0.29* | 2.69±0.22* |
| Ang-II (fmol/mg) | 4.26±0.49 | 12.44±1.03* | 5.41±0.50^#^ | 10.94±0.57*^†^ | 9.84±0.78*^†^ | 10.33±0.95*^†^ | 5.05±0.59^#&^ | 10.49±1.11* | 11.08±1.23* |

Abbreviations as in Supplementary Table 2. **P* < 0.05 vs. control group; ^#^*P* < 0.05 vs. mock group; ^†^*P* < 0.05 vs. perindopril (P) group; ^&^*P* < 0.05 vs. high-dose Ang-(1–7) (800 ng·kg^-1^·min^-1^) (A800) group.

**Full-length blots**


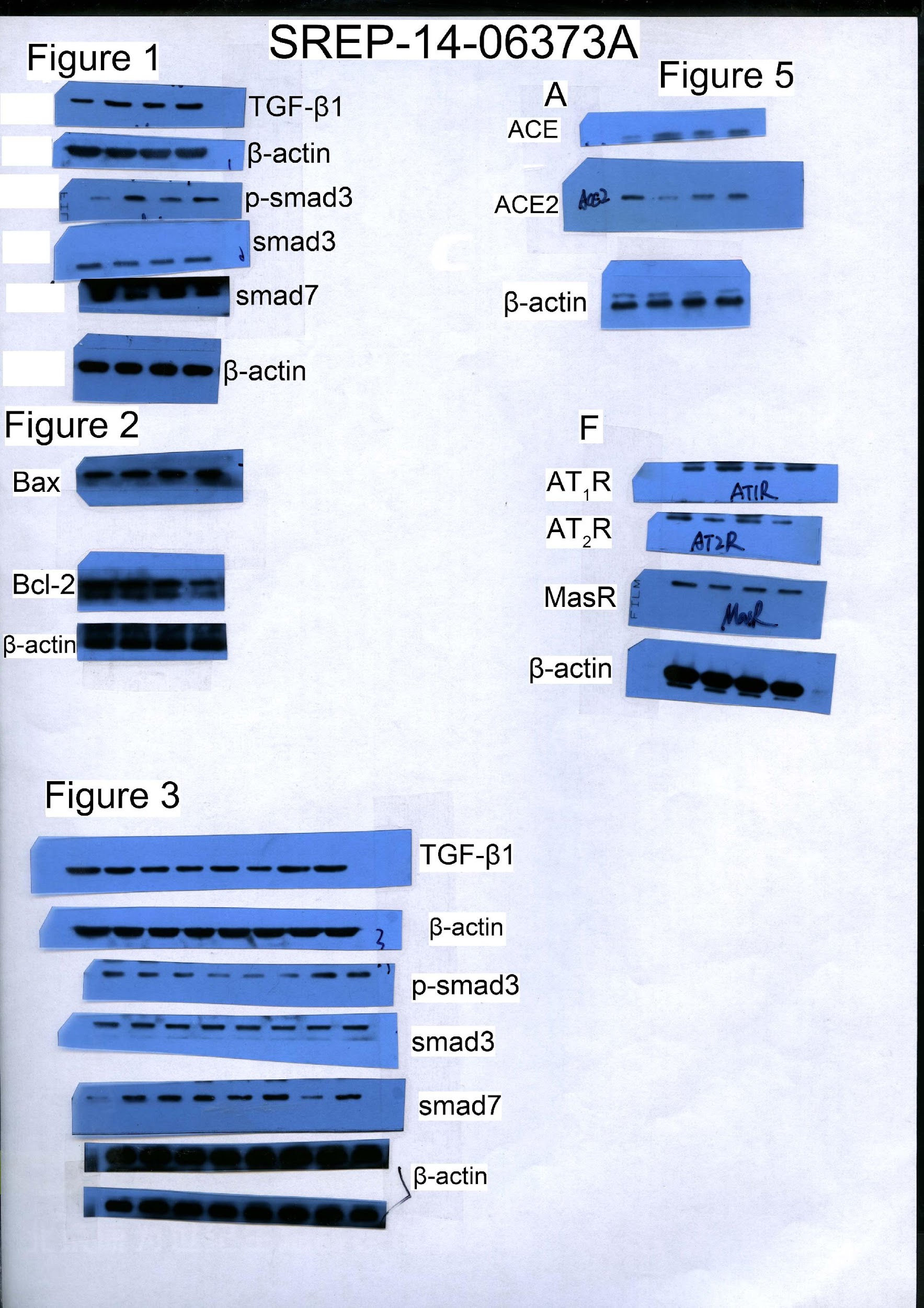


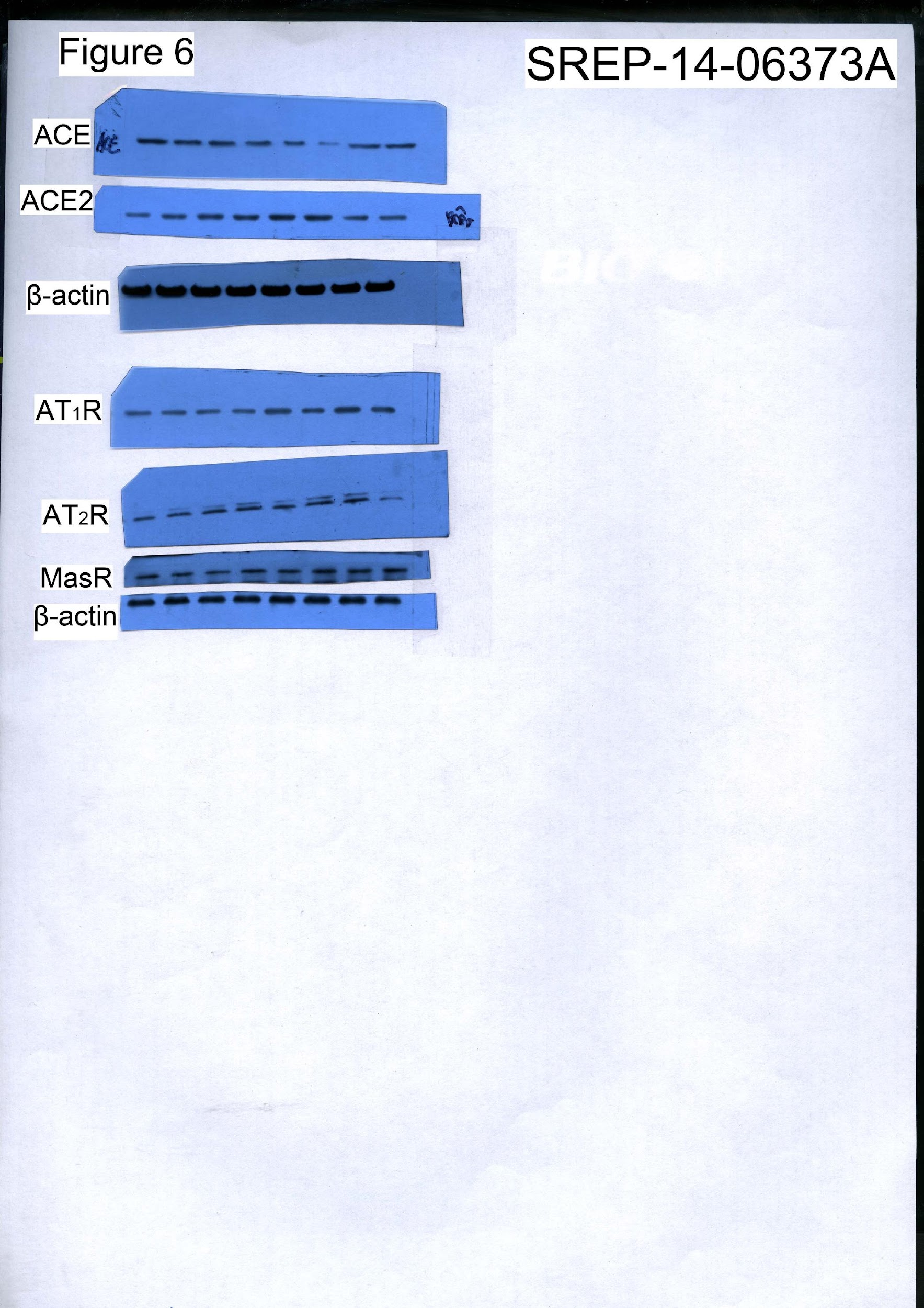

Supplement: Supplementary Information — Combined supplementary information [file srep08794-s1.docx]
